# *Yersinia pseudotuberculosis* YopH targets SKAP2-dependent and independent signaling pathways to block neutrophil antimicrobial mechanisms during infection

**DOI:** 10.1371/journal.ppat.1008576

**Published:** 2020-05-11

**Authors:** Lamyaa Shaban, Giang T. Nguyen, Benjamin D. Mecsas-Faxon, Kenneth D. Swanson, Shumin Tan, Joan Mecsas

**Affiliations:** 1 Graduate Program in Molecular Microbiology, Tufts Graduate Biomedical Sciences, Boston Massachusetts, United States of America; 2 Graduate Program in Immunology, Tufts Graduate Biomedical Sciences, Boston Massachusetts, United States of America; 3 Dept of Molecular Biology and Microbiology, Tufts University School of Medicine, Boston Massachusetts, United States of America; 4 Brain Tumor Center and Neuro-Oncology Unit, Department of Neurology, Harvard Medical School, Beth Israel Deaconess Medical Center, Boston Massachusetts, United States of America; Institut de Recherche en Santé Digestive, FRANCE

## Abstract

*Yersinia* suppress neutrophil responses by using a type 3 secretion system (T3SS) to inject 6–7 *Yersinia* effector proteins (Yops) effectors into their cytoplasm. YopH is a tyrosine phosphatase that causes dephosphorylation of the adaptor protein SKAP2, among other targets in neutrophils. SKAP2 functions in reactive oxygen species (ROS) production, phagocytosis, and integrin-mediated migration by neutrophils. Here we identify essential neutrophil functions targeted by YopH, and investigate how the interaction between YopH and SKAP2 influence *Yersinia pseudotuberculosis* (*Yptb*) survival in tissues. The growth defect of a *ΔyopH* mutant was restored in mice defective in the NADPH oxidase complex, demonstrating that YopH is critical for protecting *Yptb* from ROS during infection. The growth of a *ΔyopH* mutant was partially restored in *Skap2*-deficient (*Skap2KO*) mice compared to wild-type (WT) mice, while induction of neutropenia further enhanced the growth of the *ΔyopH* mutant in both WT and *Skap2KO* mice. YopH inhibited both ROS production and degranulation triggered via integrin receptor, G-protein coupled receptor (GPCR), and Fcγ receptor (FcγR) stimulation. SKAP2 was required for integrin receptor and GPCR-mediated ROS production, but dispensable for degranulation under all conditions tested. YopH blocked SKAP2-independent FcγR-stimulated phosphorylation of the proximal signaling proteins Syk, SLP-76, and PLCγ2, and the more distal signaling protein ERK1/2, while only ERK1/2 phosphorylation was dependent on SKAP2 following integrin receptor activation. These findings reveal that YopH prevents activation of both SKAP2-dependent and -independent neutrophilic defenses, uncouple integrin- and GPCR-dependent ROS production from FcγR responses based on their SKAP2 dependency, and show that SKAP2 is not required for degranulation.

## Author summary

Pathogenic *Yersinia* species carry a virulence plasmid encoding a type 3 secretion system that translocates 6–7 effector Yops into host cells. We demonstrate that YopH protects *Yersinia pseudotuberculosis* from neutrophil-produced reactive oxygen species (ROS) and degranulation by interfering with signaling pathways downstream of three major receptor classes in neutrophils. We show that a previously identified target of YopH, SKAP2, controls some of the pathways essential for YopH to inactivate during infection. SKAP2 is essential in mediating ROS production downstream of two major receptors; however, it is dispensable for degranulation from the three major receptors tested. Our study illustrates that YopH protects *Y*. *pseudotuberculosis* by blocking both SKAP2-dependent and independent signaling pathways that regulate several neutrophil functions.

## Introduction

The three pathogenic species of *Yersinia* that are associated with human infections are the causative agent of the pneumonic and bubonic plague, *Yersinia pestis*, and the gastrointestinal pathogens *Y*. *pseudotuberculosis* (*Yptb*) and *Y*. *enterocolitica* [1–3]. These species harbor a 70kb virulence plasmid encoding a Type 3 Secretion System (T3SS) that injects a set of 6–7 effector proteins, called Yops (Yersinia outer proteins), into mammalian cells [1, 4–7]. Yops are essential for virulence and interfere with many antimicrobial responses in immune cells, yet their functions in tissue infections are not completely understood [5, 8–11]. Infection with *Yptb* in tissues is characterized by formation of *Yptb* microcolonies that directly interface with neutrophils that have migrated to sites of infection [12–14]. Consistent with their physical proximity to *Yptb* microcolonies in tissues, neutrophils are typically the cells that are most frequently injected with Yops [15–17]. *Yptb* encodes two bacterial adhesins, Invasin and YadA, which bind either directly or indirectly to β1 integrins expressed on neutrophils [18, 19]. This binding facilitates Yop-injection into neutrophils during tissue infection, whereas in the absence of Yops, the binding via these adhesins triggers phagocytosis of *Yptb* [15, 20, 21]. In addition to integrin receptor-mediated interactions, *Yersinia* activates other receptors on neutrophils during infection, including G-protein coupled receptors (GPCRs) [22–25]. Activation of integrin receptors and GPCRs in turn leads to a variety of neutrophil antimicrobial responses including reactive oxygen species (ROS) production and proteolytic enzyme release from granule stores, also known as degranulation.

The effector, YopH, is critical for the colonization and virulence of all three pathogenic *Yersinia* species, is homologous to eukaryotic protein tyrosine phosphatases (PTPase), and is the most active of all known PTPases [5, 26–35]. Growth of a *Yptb ΔyopH* mutant is significantly restored in the absence of neutrophils, indicating that YopH targets antimicrobial functions of neutrophils [36]. In isolated human or murine neutrophils YopH inhibits zymosan-induced ROS production, calcium flux, phagocytosis, and cytokine production [36–39], and together with YopE blocks human neutrophil degranulation [40–42]. While these findings imply potential roles for YopH in modulating neutrophil functions during tissue infection, the specific signal-transduction pathway(s) that are necessary for YopH to inactivate for *Yptb* virulence in animal tissues remains unknown. To begin to address this question, some tyrosine-phosphorylated signal transduction pathway(s) disrupted by YopH during animal infection were previously identified [36]. In neutrophils isolated from spleens of infected mice, the adaptor proteins Src Kinase Associated Phosphoprotein 2 (SKAP2), PML-RARA Regulated Adaptor Molecule 1 (PRAM-1) and SH2 domain containing leukocyte protein of 76kDa (SLP-76), and the effector proteins, Vav guanine nucleotide exchange factor 1 (Vav1) and Phospholipase Cγ 2 (PLCγ2), are dephosphorylated in the presence of YopH [36]. Previous work in macrophages has shown that SKAP2 and Adhesion and Degranulation Adaptor Protein (ADAP), a homolog of PRAM-1 [43], are co-immunoprecipitated by YopH [44]. These results suggest that SKAP2-controlled pathways may be a critical target of YopH in neutrophils during tissue infection.

SKAP2 is a cytosolic adaptor protein comprised of a N-terminal coiled-coil dimerization domain region, a proximal pleckstrin homology domain (PH), an interdomain containing two tyrosine-based signaling motifs and a C-terminal Src homology 3 (SH3) domain that mediates its binding to PRAM-1 and ADAP [45–48]. SKAP2 is a substrate for Scr kinases, and was recently found to be essential in regulating TNFα–, selectin- and CXCL1-mediated integrin activation [49]. In addition, SKAP2 regulates neutrophil functions including migration to sites of sterile inflammation, neutrophil extracellular trap (NET) formation, and integrin-mediated ROS production [49].

ROS generated by the activated NADPH oxidase complex occurs after stimulation of various surface receptors, including integrin, GPCR, and Fc receptors [49–55] that triggers assembly and activation of the NADPH oxidase complex at the plasma or phagosomal membrane. The gp91^phox^ subunit is required for the assembly of a functional NADPH oxidase complex and subsequent superoxide production [56]. ROS can act directly as an antimicrobial agent by causing oxidative damage to DNA molecules and destroying pathogen membrane structures by lipid peroxidation. It can also activate other neutrophil antimicrobial functions such as phagocytosis [57–59], cytokine release [60–62], NET formation [63–65], and degranulation [57, 66].

Here we investigate whether YopH is required to prevent ROS production during *Yptb* infection of mouse tissues and whether SKAP2 must be inactivated for a Δ*yopH* mutant to survive and thus is an important YopH target. We find that while inactivation of SKAP2-regulated pathways is a key function of YopH, YopH has additional functions independent of SKAP2 that enable further inhibition of both neutrophil ROS production and degranulation. Our studies also shed light on SKAP2 function, demonstrating its essential role in neutrophil ROS activation after integrin or GPCR-stimulation, but not in degranulation or migration of neutrophils to infected tissues sites.

## Results

### Growth of a *ΔyopH* mutant is restored in mice lacking a functional NADPH oxidase complex and YopH is sufficient to block ROS production from murine neutrophils

**Fig 1:**
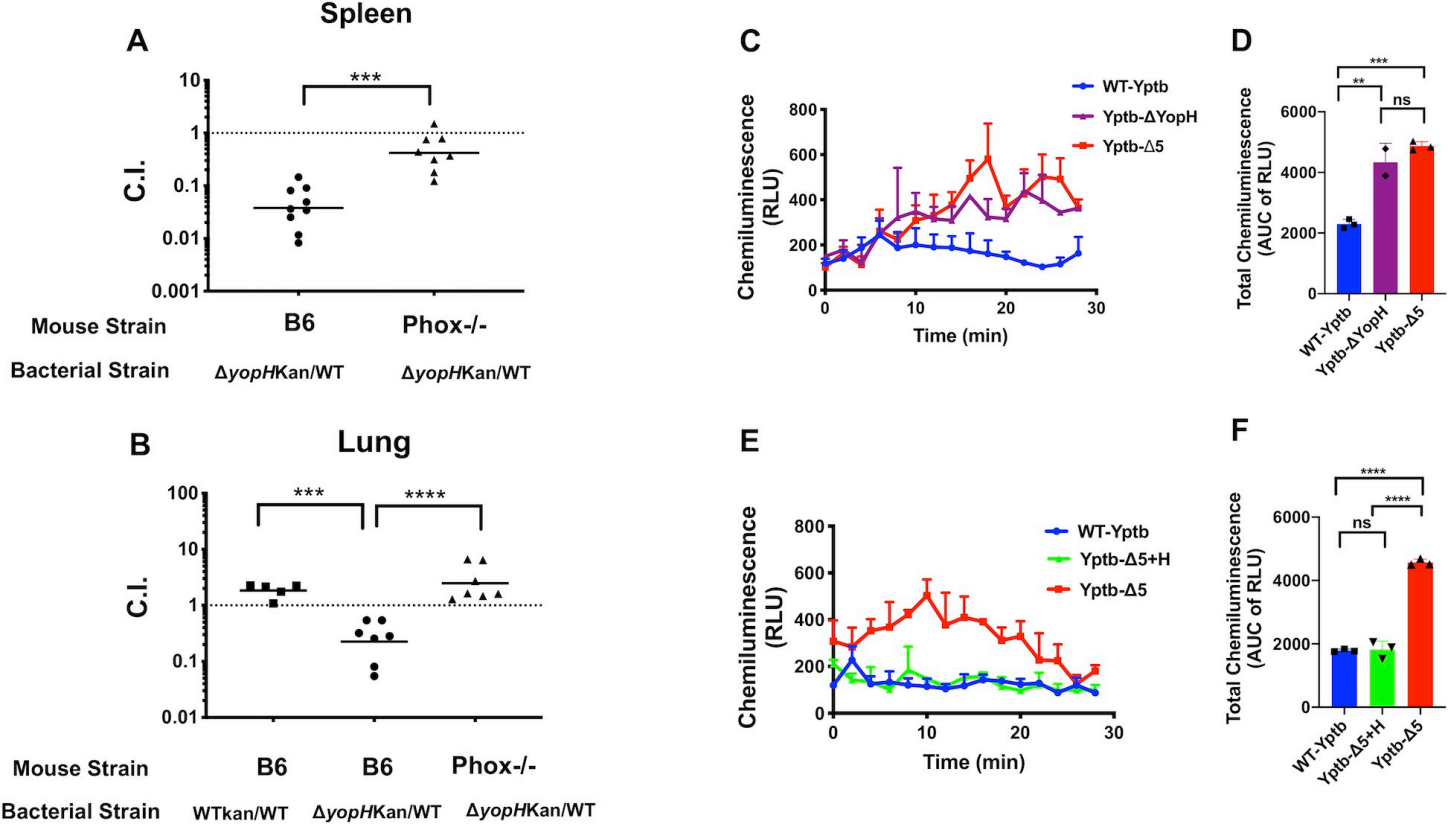
The growth of a *ΔyopH* mutant is restored in absence of ROS *in vivo* and YopH is necessary and sufficient to block extracellular ROS produced by BM neutrophils *in vitro*. (A-B) Female C57BL/6J or C57BL/6J *gp91*^*phox-/-*^ mice were inoculated with a 1:1 mixture of IP2666 WT-*Yptb* and Δ*yopH*-Kan^R^ (A) intravenously with 1x10^3^ CFU or (B) intratracheally with 5x10^3^ CFU. Spleens and lungs were collected, weighed, homogenized, and plated for CFU two days post- infection. The competitive indices (C.I.) were determined by patching 100 colonies on L-Irgasan and L-Irgasan+Kanamycin plates. Each symbol represents an individual mouse; horizontal bars represent the geometric mean. (C-F) BM neutrophils were infected at a MOI of 20:1 with (C-D) WT-*Yptb*, *ΔyopH*, or Δ5 (a mutant lacking 5 effector Yops, H, E, O, M, J), (E-F) WT-*Yptb*+pBAD, Δ5+YopH (a strain expressing only YopH at native levels from an arabinose inducible plasmid),or Δ5+pBAD, and monitored for extracellular ROS production using isoluminol chemiluminescence assay. (D and F) Total chemiluminescence was determined by computing the area under the curve (AUC) using GraphPad Prism version 7. Mean ±SD of a representative experiment from 2–3 independent experiments done in 2–3 replicates is presented. Statistical significance was calculated using (A) the Mann-Whitney t-test, (B) one-way ANOVA followed by Tukey’s Post-test after log_10_ transformation of the data, or (D, F) one-way ANOVA followed by Tukey’s Post-test for area under the curve (AUC).

Our previous work [36] demonstrated that several signaling proteins that are important for ROS production downstream of integrin signaling, including SKAP2, PRAM-1, SLP-76, Vav1, and PLCγ2 [49–54], were hypo-phosphorylated in YopH-injected neutrophils isolated from infected spleens. However, whether YopH plays a role in supporting *Yptb* growth during infection by suppressing ROS production is unknown. To test this, C57BL/6J and *gp91^phox-/-^* mice were infected intravenously (I.V.) or intratracheally (I.T.) with an equal mixture of IP2666 WT-*Yptb* and a *ΔyopH* mutant. Two days post-infection spleens and lungs were harvested and the competitive index (C.I.) was determined. By contrast with previous work on *ΔyopE* [67], the *ΔyopH* mutant competed significantly better in the spleens and lungs of *gp91*^*phox-/-*^ mice than C57BL/6J mice (Fig 1A and 1B). The total CFU of *Yptb* recovered from *gp91*^*phox-/-*^ mice were comparable to numbers recovered from C57BL/6J mice, but the numbers of the recovered *ΔyopH* mutant rose significantly in the *gp91*^*phox-/-*^ mice (S1A Fig). These results support the idea YopH inactivates one or more pathways leading to the generation of ROS and thereby protects *Yptb* from superoxide-mediated killing during tissue infection.

To examine the ability of YopH to block ROS production in neutrophils, extracellular ROS production was measured after *Yptb* infection of neutrophils isolated from bone marrow (BM) of BALB/c mice (S1B Fig). BM neutrophils were infected with WT-*Yptb*, *ΔyopH*, Δ*5* (a mutant lacking 5 effector Yops, H, E, O, M, J) or Δ*5+*pYopH, a strain expressing only YopH at native levels from an arabinose inducible plasmid (S1 Table). There were no differences in amount of YopH secretion between Δ*5+*pYopH and WT-*Yptb* (S2 Fig). YopH was necessary to block ROS production induced by WT-*Yptb* infection (Fig 1C and 1D) because infection with the *ΔyopH* strain generated high levels of ROS that were equivalent to infection with the Δ*5* strain (Fig 1D). In addition, YopH was the only Yop needed to block ROS released by BM neutrophils (Fig 1E and 1F) because infection with Δ*5+*pYopH blocked ROS production as much as WT-*Yptb* infection (Fig 1F). Combined, these results indicate that YopH blocks production of extracellular ROS by neutrophils induced by *Yptb* infection.

### Growth of a Δ*yopH* mutant in the spleen is partially restored in the absence of SKAP2 and is further enhanced in neutropenic mice

**Fig 2:**
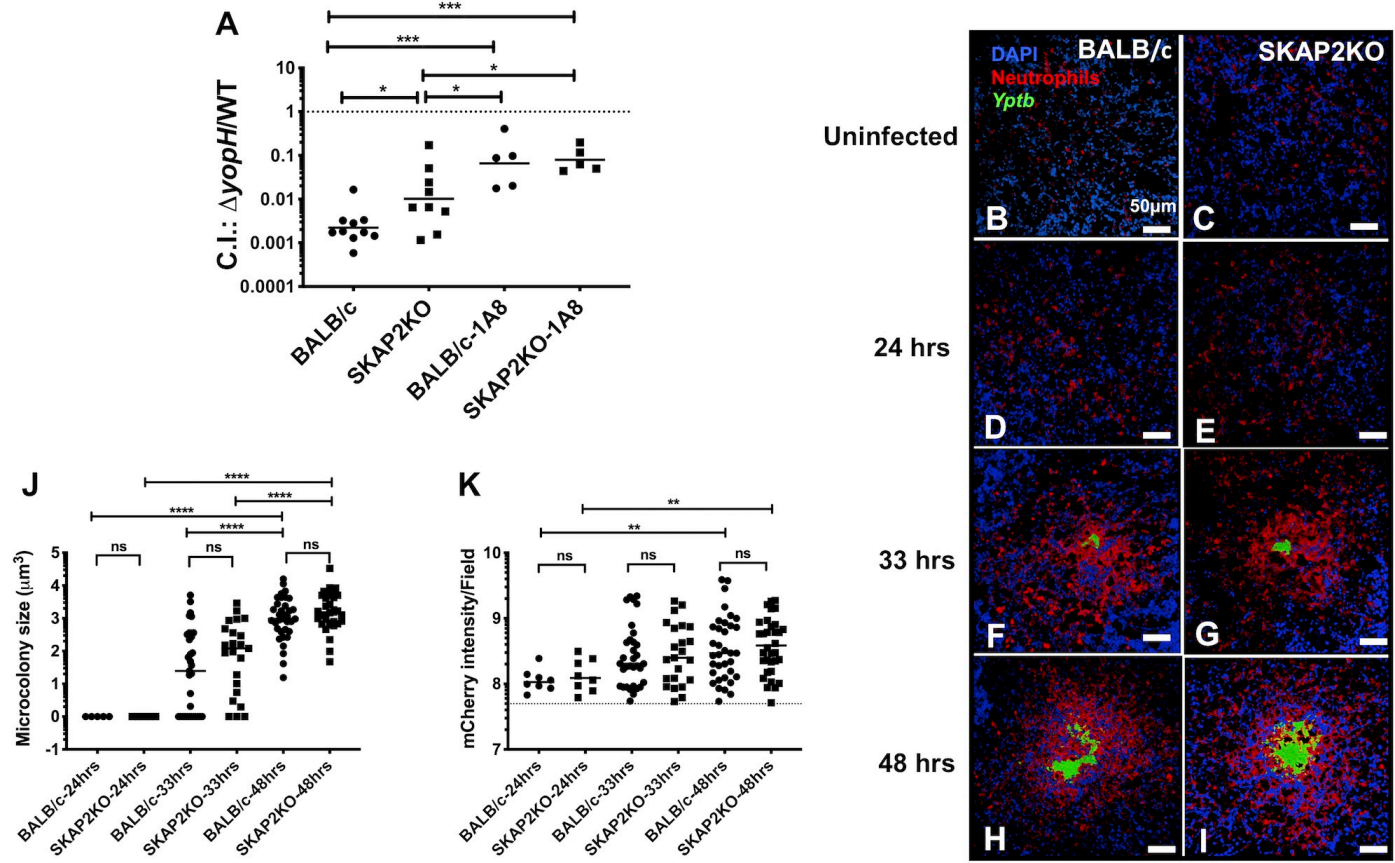
*Skap2KO* neutrophils cluster to *Yptb* microcolonies and the growth of a *ΔyopH* mutant is partially restored in competition with WT-*Yptb* in *Skap2KO* mice. (A) WT-BALB/c and *Skap2KO* mice were injected with 1A8 or an isotype control antibody and 16 hrs later I.V. infected with an equal mixture of 1x10^3^ CFU of IP2666 WT-*Yptb* and Δ*yopH-*Kan^R^. Spleens were collected three days post-infection, weighed, homogenized, and plated for CFU on selective and non-selective agar. The number of bacteria recovered from selective and non-selective plates was used to determine the C.I. Each dot represents a mouse; horizontal bars represent the geometric mean. Significance was calculated using 2-way ANOVA followed by Tukey’s Post-test of log_10_ transformed values, with only significant values shown. The results are a composite of three experiments. (B-I) BALB/c and *Skap2KO* mice were (B-C) uninfected or (D-I) I.V. infected with 10^3^ CFU of IP2666 WT-*Yptb*-GFP (green), and sacrificed at (D-E) 24, (F-G) 33, or (H-I) 48 hours post-infection. Frozen sections of spleens were stained using an anti-Ly6G antibody (neutrophils-red) and DAPI (nuclei-blue) and visualized by fluorescence confocal microscopy at 40X magnification. (J-K) Using Volocity software, the size of the (J) bacterial microcolony was determined by generating the summed volume of the individual GFP signal and the (K) number of neutrophils recruited was determined by detecting the signal intensity of the mCherry channel. Each dot represents a microcolony or an area of clustered neutrophils. The horizontal solid bars represent the median, and (K) the dotted line represents the median neutrophil signal intensity in uninfected controls of BALB/c and *Skap2KO* mice. Scale bars: 50 μm. Statistical significance was determined (J-K) using Mann-Whitney for comparing BALB/c and *Skap2KO* at each time point and Kruskal-Wallis for comparisons between the three time points for each mouse genotype. The results are a composite of 2–3 mice from two independent experiments.

SKAP2 is essential for integrin-stimulated ROS production in neutrophils [49], is dephosphorylated in neutrophils injected with YopH during tissue infection [36], and associates with YopH in macrophages [44]. These observations, combined with the observations that *Yptb* binds to neutrophils via integrins [15, 20, 68, 69] and that YopH blocks ROS production (Fig 1), led us to evaluate whether SKAP2 influences *Yptb* survival in the presence and absence of YopH. BALB/c (WT) and BALB/c-*Skap2KO* (*Skap2KO*) mice were co-infected I.V. with an equal mixture of WT-*Yptb* and a Δ*yopH* mutant. Three days post-infection spleens were collected, and the C.I. was determined. The Δ*yopH* mutant colonized *Skap2KO* mice almost 10-fold more efficiently compared to WT mice while overall bacterial burdens were similar (Fig 2A, S3A Fig), suggesting that SKAP2 may function in a YopH sensitive pathway(s) that serves to limit growth of a Δ*yopH* mutant *in vivo*. Although, *Skap2KO* mice have normal levels of circulating neutrophils [70], their extravasation into tissues occurs at significantly lower rates in a sterile model of inflammation [49]. Thus, it was possible that the improved survival of the *ΔyopH* mutant in the *Skap2KO* mice was due to reduced rates of neutrophil recruitment to infected sites. Analysis of the kinetics of neutrophil recruitment in the spleens of *Yptb*-infected WT and *Skap2KO* mice revealed no differences in the recruitment of neutrophils and bacterial burdens were comparable between the WT and *Skap2KO* mice at the tested time points (Fig 2B–2K, S3B Fig). Collectively, these results support the idea that the restoration of Δ*yopH* mutant growth in *Skap2KO* mice (Fig 2A) is due to a functional defect in *Skap2*-deficient neutrophils that increases the survival of a Δ*yopH* mutant. This suggests that SKAP2 mediates signaling from receptors stimulated by *Yptb* infection and participates in a subset of relevant antimicrobial neutrophil functions normally targeted by YopH.

The Δ*yopH* mutant was still defective for survival compared to WT-*Yptb* in *Skap2KO* mice suggesting that *Skap2KO* neutrophils may retain some killing activities against the Δ*yopH* mutant. To determine whether YopH targets pathways, which are independent of SKAP2, in neutrophils, additional cohorts of mice from each genotype were subjected to 1A8 antibody-mediated depletion of neutrophils. Growth of the Δ*yopH* mutant was significantly higher in neutrophil-depleted *Skap2KO* mice compared to *Skap2KO* mice (Fig 2A), indicating that *Skap2KO* neutrophils retain some antimicrobial activities and that YopH targets additional SKAP2-independent pathways in neutrophils. Notably, *ΔyopH* growth was comparable in neutrophil-depleted WT and neutrophil-depleted *Skap2KO* mice, highlighting that the lack of SKAP2 activity in other innate immune cells does not enhance survival of the Δ*yopH* mutant in the absence of neutrophils. Thus, YopH targets both SKAP2-dependent and SKAP2-independent antimicrobial processes.

### YopH blocks SKAP2-dependent and -independent ROS production by neutrophils

**Fig 3:**
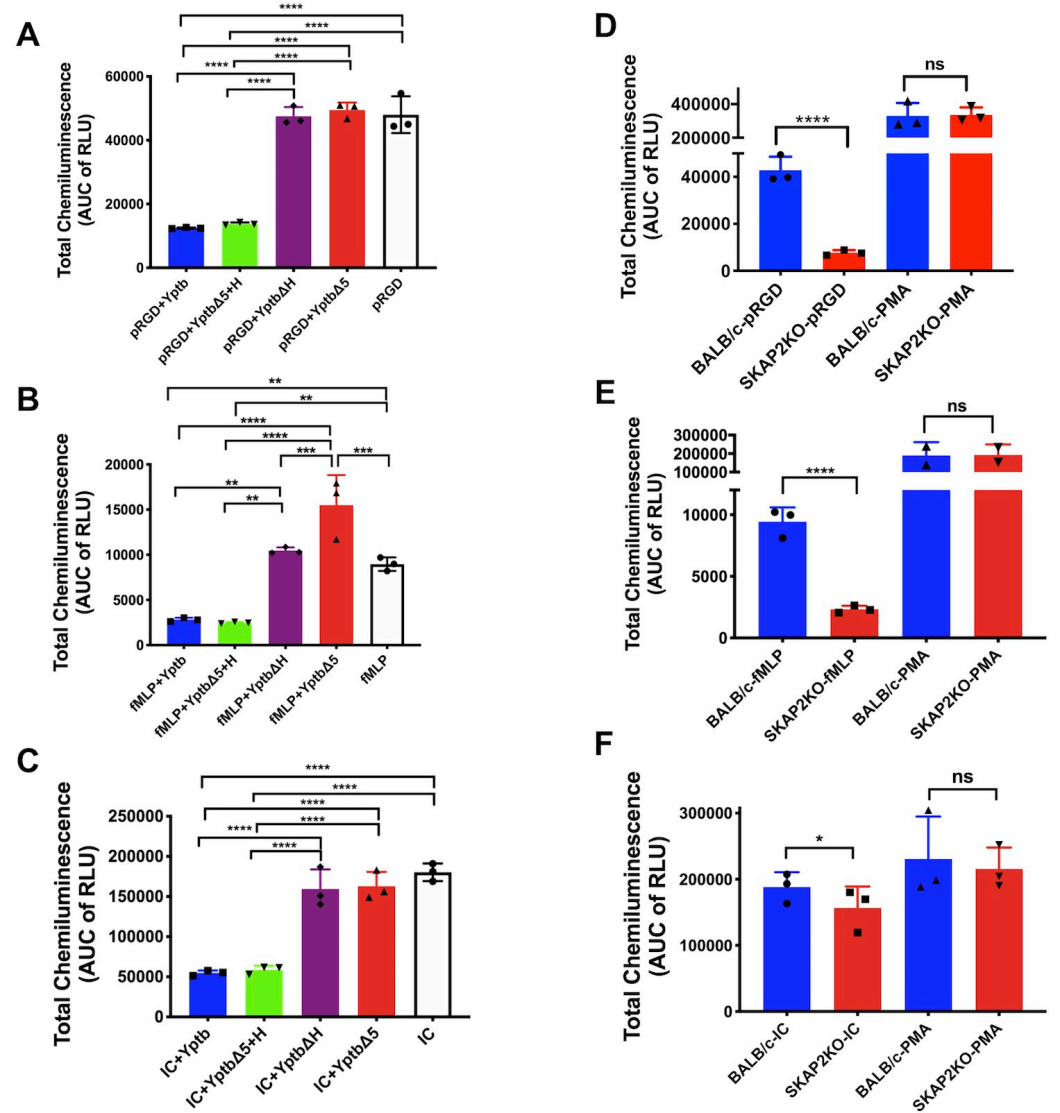
YopH is necessary to block ROS from all three receptors while *Skap2KO* neutrophils fail to produce ROS following integrin receptor and GPCR but not FcγR stimulation. Respiratory burst of (A-C) WT BM neutrophils that were either uninfected or infected with IP2666 WT-*Yptb*, *Yptb-*Δ*5+*pBAD, *Yptb-*Δ*5*+pYopH or *Yptb-*Δ*H* at a MOI of 20:1, or (D-F) BM neutrophils from WT and *Skap2KO* mice was measured using an isoluminol chemiluminescence assay. BM neutrophils were added to (A, D) poly-RGD coated surface for 30 min, (B, E) primed with 10μg/ml LPS for 20 min followed by stimulation with 1μM fMLP for 10 min, or (C, F) added to IC coated surface for 30 min and monitored for extracellular ROS production. (D-F) PMA (1μM) was added to WT and *Skap2KO* neutrophils as a positive control. Total chemiluminescence was determined by computing the area under the curve for the duration of the experiment for each condition after subtracting the uninfected, unstimulated control plated on FBS. Statistical significance was calculated using (A-C) one-way ANOVA followed by Tukey’s Post-test or (D-F) Student t-test. Only statistically significant comparisons are shown. Results presented are the mean ± SD of triplicate measurements. Data is a representative of at least three independent experiments done in triplicates.

Since Δ*yopH* growth was restored in *gp91^phox-/-^* mice we evaluated the ability of YopH to block ROS production downstream of three major receptor classes (integrin receptors, G- protein coupled receptor (GPCR) and FcγR) known to activate the NADPH oxidase complex, two of which may be stimulated during *Yptb* infection [18, 19, 22–25]. BM WT neutrophils were infected with WT-*Yptb*, *ΔyopH*, Δ*5*, or Δ*5+*pYopH and stimulated with poly-RGD (a polymer of arginine-glutamate-aspartate) to trigger integrin receptors, primed with *E*. *coli* lipopolysaccharide (LPS) and then stimulated with formyl-methionyl-leucyl-phenylalanine (fMLP) to trigger GPCR, or stimulated with IgG immune complex (IC) to trigger Fcγ receptor (FcγR) (Fig 3A–3C) [49, 55, 71–76]. In all cases, WT-*Yptb* and Δ*5*+pYopH inhibited ROS production similarly demonstrating that YopH was sufficient for ROS inhibition. In addition, high levels of ROS were detected in BM neutrophils infected with Δ5 or Δ*yopH* (Fig 3A–3C) demonstrating that YopH was also necessary for ROS inhibition when other Yops are present. Unlike stimulation with integrin or Fcγ receptors (Fig 3A and 3C), ROS production from Δ*yopH*-infected from BM neutrophils that were exposed to LPS and fMLP was less than Δ*5*-infected cells, indicating that another Yop acts to partially reduce ROS production in the absence of YopH downstream of GPCR stimulation (Fig 3B). Further, ROS production after infection with Δ5 and exposure to LPS and fMLP was significantly higher than after exposure to LPS and fMLP alone (Fig 3B). This suggests that infection with Δ5 augments ROS production through activation of other receptors, possibly due to bacteria-mediated crosslinking of and signaling by integrin receptor in the absence of Yops (Fig 1C–1F) [18, 19].

Given our findings that *Skap2KO* mice retain some killing activities against the Δ*yopH* mutant (Fig 2A), we next tested the ability of *Skap2KO* neutrophils to produce ROS when stimulated through integrin receptor, GPCR and FcγR (Fig 3D–3F). *Skap2KO* neutrophils failed to generate high levels of ROS after poly-RGD and LPS + fMLP exposure (Fig 3D and 3E, S4A–S4D Fig), but stimulation of FcγR in *Skap2KO* neutrophils resulted in high levels of extracellular ROS, amounting to 80–90% of the ROS produced by WT neutrophils (Fig 3F, S4E and S4F Fig). To verify that defects in ROS production resulted from a receptor-mediated signaling defect and not an inability of *Skap2KO* neutrophils to activate the NADPH oxidase complex, WT and *Skap2KO* neutrophils were stimulated with phorbol myristate acetate (PMA), which bypasses receptor-mediated activation and directly activates protein kinase C (PKC), to trigger assembly of the NADPH oxidase complex [55, 73, 74]. Indeed, PMA-stimulated WT and *Skap2KO* neutrophils produce equivalent levels of ROS (Fig 3D–3F). Together, these results indicate that a functional NADPH oxidase complex can assemble in *Skap2KO* neutrophils, but that SKAP2 signaling is required for integrin receptor and GPCR-mediated ROS production in neutrophils. Since these two receptors may be stimulated during infection with *Yptb*, these results suggest that the inability of *Skap2KO* neutrophils to generate ROS contributes to the partial growth restoration of Δ*yopH* observed during *in vivo* infections (Fig 2A). While FcγR is unlikely to be activated by *Yptb* during infection of a naïve host, this result nonetheless raises the possibility that neutrophils have other receptors that may recognize *Yptb*, signal and function independently of SKAP2, leading to ROS generation in the absence of YopH. This superoxide production could potentially contribute to partial elimination of the Δ*yopH* mutant in *Skap2KO* mice.

### YopH reduces phosphorylation of signaling proteins upon FcγR-stimulation independent of SKAP2

**Fig 4:**
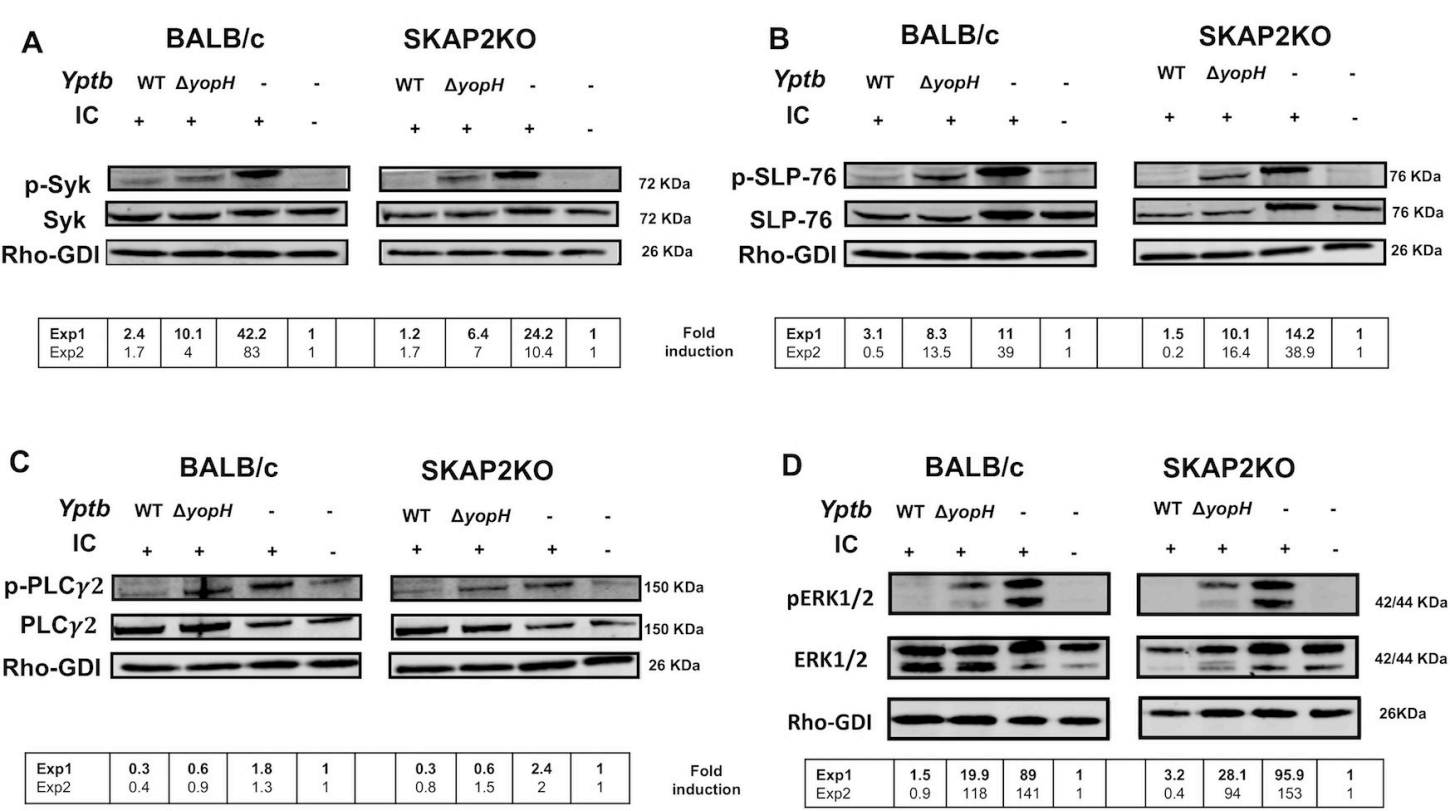
YopH dephosphorylates SKAP2-independent signaling proteins downstream of FcγR receptor. WT-BALB/c and *Skap2KO* BM neutrophils were uninfected or infected with YPIII WT-*Yptb* or *ΔyopH* then stimulated on IC coated surface for 5 min. Lysates were immunoblotted with (A) anti-Syk pY352 striped and then reprobed with anti-Syk, (B) anti-SLP-76 pY128 striped and then reprobed with anti-SLP-76, or (C) anti- PLCγ2 pY1217 striped and then reprobed with anti- PLCγ2, or (D) anti-ERK1/2 (pThr202/pTyr204) striped and reprobed with anti-ERK1/2. Anti-Rho-GDI was used as a loading control for all blots. Fold induction was calculated by dividing the normalized phospho-tyrosine signal by the unstimulated and uninfected control of each genotype. Blots shown are a representative of 2 independent experiments. Fold induction values from each experiment is shown in the lower panel with blots from experiment 1 (Exp1) presented.

**Fig 5:**
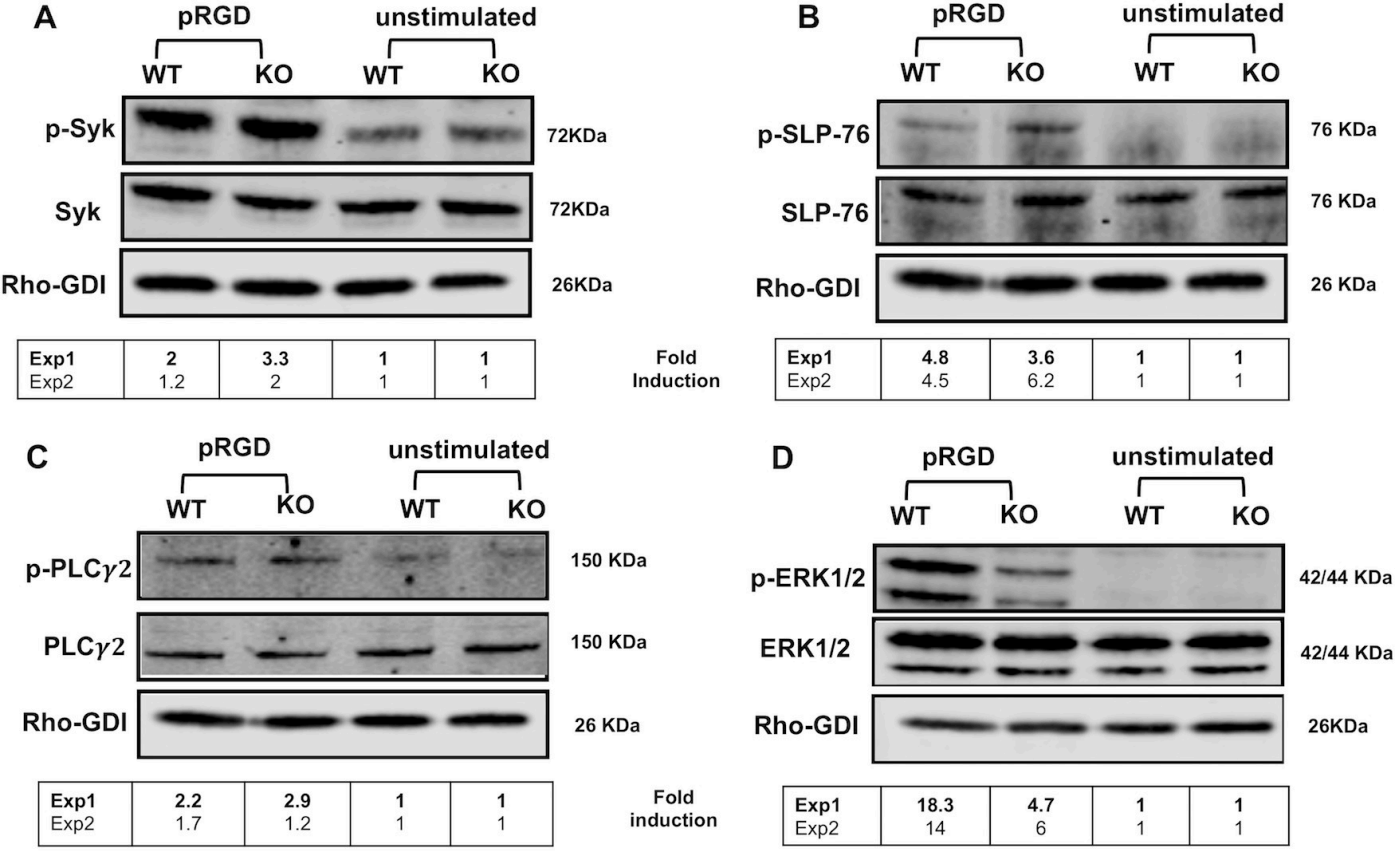
SKAP2 affects ERK1/2 signaling downstream of integrin receptor stimulation in neutrophils. WT and *Skap2KO* BM neutrophils were unstimulated on a FBS coated surface or stimulated on poly-RGD coated surface for 10 min, and lysates were immunoblotted with (A) anti-Syk pY352, striped and then reprobed with anti-Syk, (B) anti-SLP-76 pY128, striped and then re-probed with anti-SLP-76, (C) anti-PLCγ2 pY1217, striped, and then reprobed with anti- PLCγ2, or (D) anti-ERK1/2 (pThr202/pTyr204) striped and reprobed with anti-ERK1/2. Anti-Rho-GDI was used as a loading control for all blots. Fold induction was calculated by dividing the normalized phospho-tyrosine signal by the unstimulated control of each genotype. Blots shown are a representative of 2 independent experiments. Fold induction values from each experiment is shown in the lower panel with blots from experiment 1 (Exp1) presented.

Since YopH is sufficient to block FcγR-mediated ROS production (Fig 3C) but *Skap2KO* neutrophils produce ROS downstream of FcγR triggering by IC (Fig 3F), there remains an intact SKAP2-independent ROS-producing pathway downstream of FcγR that is targeted by YopH. We therefore evaluated whether YopH reduced tyrosine phosphorylation of signaling molecules important for ROS production after FcγR stimulation, namely Syk (pY352), SLP-76 (pY128), PLCγ2 (pY1217) and ERK1/2 (pThr202/pTyr204) [53, 54, 80]. All were robustly phosphorylated in both WT and *Skap2KO* neutrophils after exposure to IC (Fig 4A–4D) indicating that these signaling proteins are activated upstream and/or independent of SKAP2. However, YopH reduced tyrosine phosphorylation of all these proteins (Fig 4A–4D) showing that YopH can interfere with ROS-generating pathways independent of SKAP2. In comparison, we evaluated whether phosphorylation of Syk, SLP-76, PLCγ2, and ERK1/2 required SKAP2 after integrin stimulation, since *Skap2KO* neutrophils failed to produce ROS downstream of the integrin receptor (Fig 3D). SKAP2 was not required for the phosphorylation of Syk (pY352), SLP-76 (pY128) and PLCγ2 (pY1217) but was required for maximal phosphorylation of ERK1/2 (pThr202/pTyr204) (Fig 5A–5D). WT and *Skap2KO* BM neutrophils had low levels of SKAP1 indicating that the loss of SKAP2 was not compensated by overexpression of its homologue SKAP1 (S5 Fig). Therefore, SKAP2 acts downstream or independently of Syk, SLP-76 and PLCγ2 phosphorylation after integrin activation, but is important for relaying signals to activate ERK1/2, which is required for NADPH oxidase complex activity [49, 77–80]. Furthermore, YopH can interrupt both SKAP2-dependent and independent signaling pathways.

### YopH targets degranulation downstream of integrin receptor, GPCR and FcγR, and degranulation is SKAP2-independent

**Fig 6:**
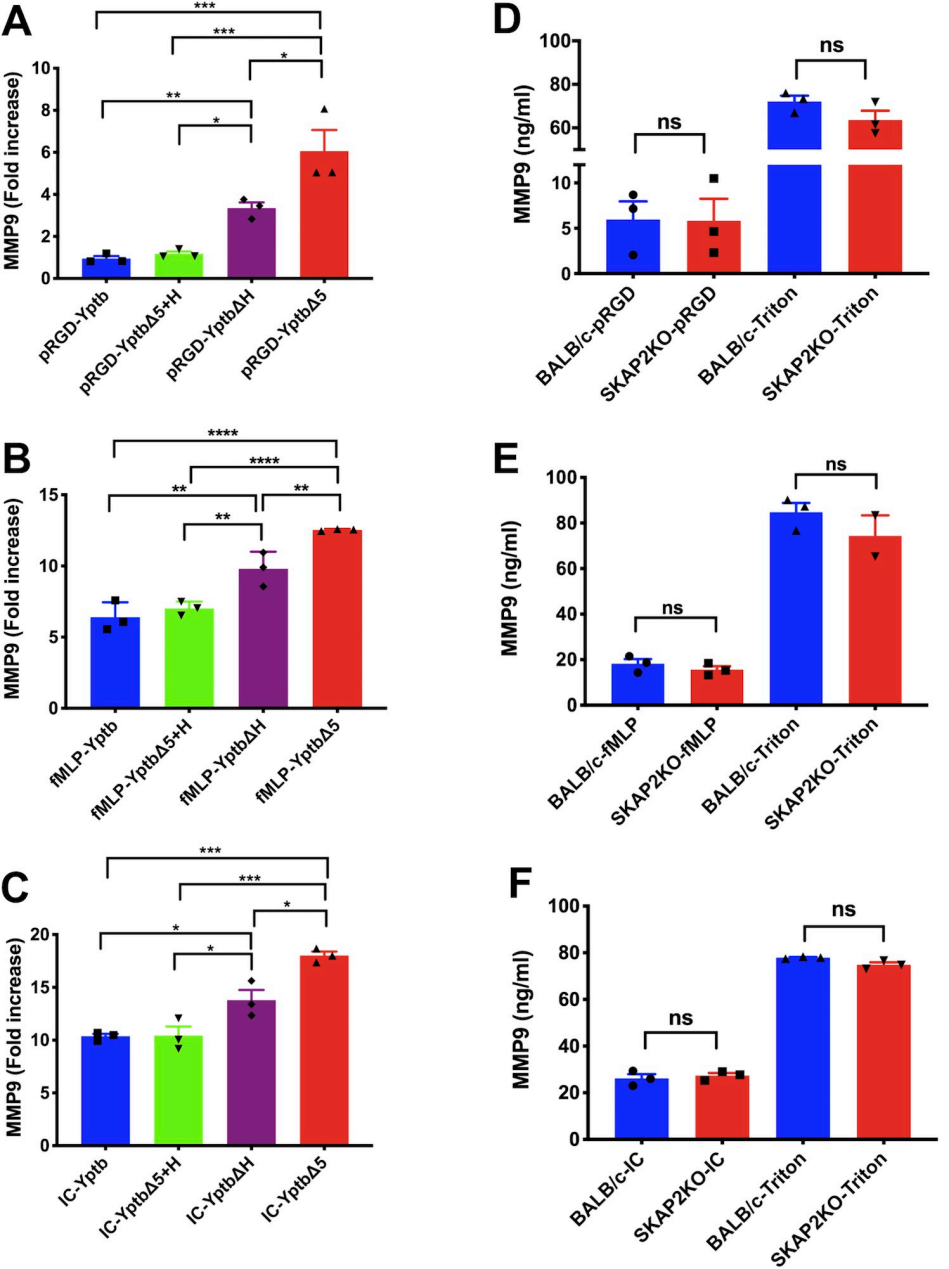
YopH reduces tertiary granule release, while SKAP2 is dispensable for tertiary granule release in neutrophils. Tertiary degranulation of (A-C) WT BM neutrophils that were simultaneously infected with WT-*Yptb*, *Yptb-*Δ*5*+pBAD, *Yptb-*Δ*5*+pYopH or *Yptb-ΔyopH* at a MOI of 20:1 or of (D-F) WT and *Skap2KO* BM neutrophils stimulated for 3 hrs by (A, D) plating on poly-RGD, (B, E) priming with LPS for 20 minutes and treating with 1 μM fMLP, or (C, F) plating on IC. Cell free supernatants were analyzed for MMP-9 by ELISA with background subtraction of the unstimulated controls (FBS-plated controls). (A-C) Data for infections are expressed as fold increase relative to uninfected, unstimulated control samples from FBS coated plates. (D-F) Amount of MMP-9 (ng/ml) for WT and *Skap2KO* BM neutrophils with background subtraction of the unstimulated (FBS-plated) controls are reported. Triton X-100-treated wells were used to determine the total granule content of WT and *Skap2KO* BM neutrophils. The data represent the means ± SEM from 3 independent experiments done in 2–3 replicates. Statistical significance was calculated using (A-C) one-way ANOVA followed by Tukey’s Post-test or (D-F) Student t-test.

To investigate the ability of YopH to inhibit additional antimicrobial activities and evaluate whether these activities are controlled by SKAP2, we assessed YopH-dependent inhibition of degranulation in WT and *Skap2KO* neutrophils. In human and murine neutrophils, YopH partially contributes to the inhibition of neutrophil degranulation as mutants infected with either Δ*yopH* or Δ*yopE* are partially defective in degranulation [40, 41]. The ability of YopH to prevent tertiary degranulation was tested by infecting WT BM neutrophils with WT-*Yptb*, *ΔyopH*, Δ*5* or Δ5+pYopH and stimulating them with poly-RGD, fMLP, or IC (Fig 6A–6C). The amount of matrix metalloproteinase-9 (MMP-9), a component of tertiary granules, released in the cell-free supernatants was quantified by ELISA. Western blot analysis with anti-Rho-GDI antibody confirmed that the observed release of MMP-9 was not due to neutrophil lysis (S6A Fig). Degranulation was completely inhibited by WT-*Yptb* after stimulation with poly-RGD (Fig 6A) compared to uninfected controls, and was partially blocked by WT-*Yptb* after exposure to LPS+fMLP or IC (Fig 6B and 6C), indicating the combined actions of the Yops are insufficient to halt degranulation under receptor stimulation (Fig 6B and 6C). Interestingly, degranulation was higher when cells were stimulated with either fMLP or IC and infected with Δ*5* compared to stimulation of receptors in the absence of infection (S6B Fig), suggesting that simultaneous Δ*5* binding via integrin receptor and stimulation of GPCR or FcγR enhances degranulation levels. YopH was sufficient to block tertiary granule release as Δ*5*+pYopH-infected neutrophils released MMP-9 to the same level as WT-*Yptb* downstream of integrin, GPCR, and FcγR (Fig 6A–6C). Consistent with previous results [40, 41], significantly more degranulation was observed after infection with Δ*yopH* compared to WT-*Yptb*, but significantly less compared to infection with Δ5 (Fig 6A–6C). This demonstrates that YopH plays a role in reducing degranulation in murine neutrophils but is not the sole factor involved. Of note, SKAP2 was dispensable for MMP-9 release with approximately 6 ng/ml, 20 ng/ml and 25 ng/ml MMP-9 released after integrin, GPCR and FcγR stimulation, respectively (Fig 6D–6F). Supernatants of PMA-stimulated neutrophils showed 50% degranulation compared to triton X-100 treatment (S6C Fig). Combined, these results indicate that SKAP2 is dispensable for tertiary degranulation and suggests that SKAP2KO neutrophils may control growth of the Δ*yopH* mutant through degranulation.

### *Skap2KO* neutrophils are defective for phagocytosis of *Yptb*

**Fig 7:**
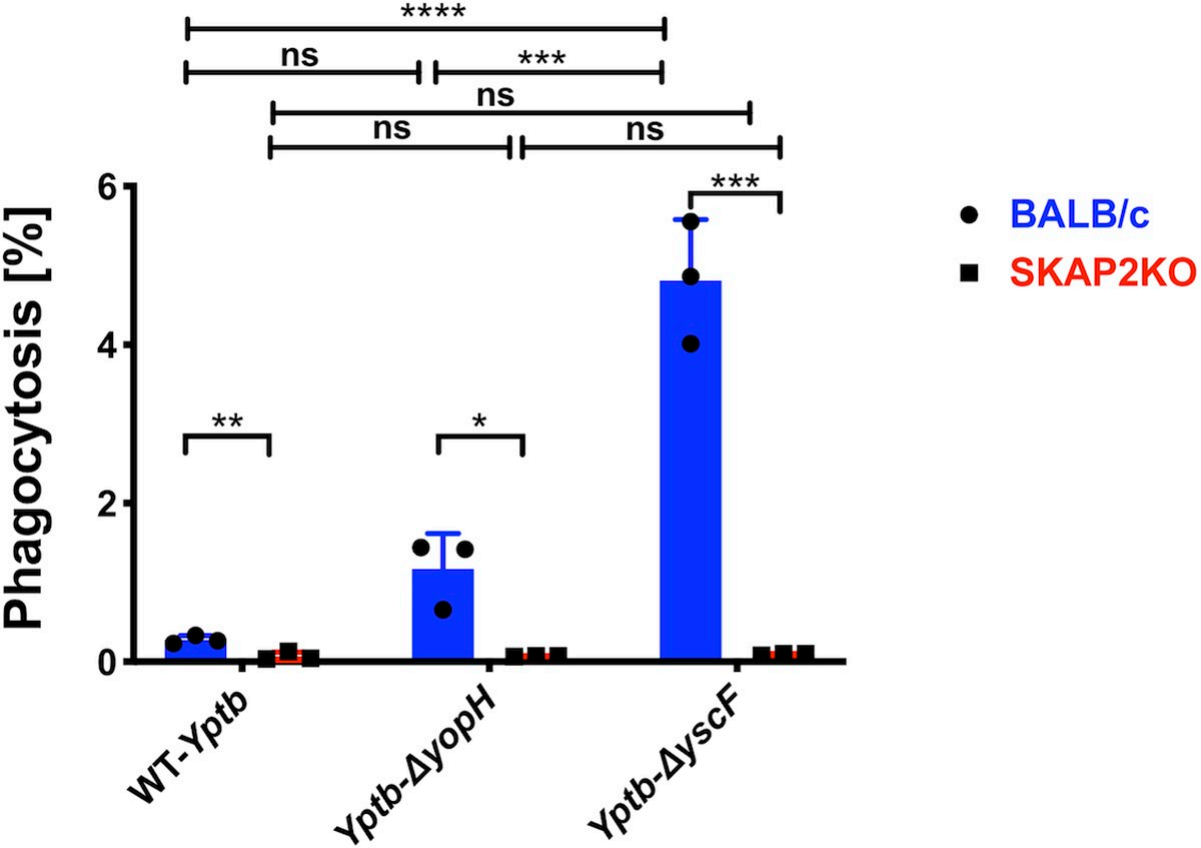
*SKAP2KO* neutrophils are defective in phagocytosis of a T3SS *Yptb* mutant. WT-BALB/c and *Skap2KO* BM neutrophils were infected with YPIII WT-*Yptb*, *ΔyopH*, and *ΔyscF* at MOI of 10:1 for 30 min. A gentamicin protection assay was used to determine the percentage of phagocytosed bacteria. Bars indicate mean of triplicate assays ± SD. Individual dots show each replicate. Experiment shown is a representative of 3 independent experiments. Statistical significance was calculated using a Student’s t-test to compare WT and *SKAP2KO* neutrophils and one-way ANOVA followed by Tukey’s Post-test for comparisons between different infections of each neutrophil genotype.

Finally, given that YopH participates in the ability of *Yersinia* to resist phagocytosis in isolated human neutrophils [29, 37, 39, 81, 82], we evaluated the ability of *Skap2KO* neutrophils to phagocytose WT-*Yptb*, *ΔyopH*, or a strain defective for the type 3 secretion system, *ΔyscF*. Using a gentamicin protection assay, we found that all three strains were phagocytosed at a similar, low level by *Skap2KO* BM neutrophils and at significantly lower levels compared to that of WT BM neutrophils (Fig 7). This suggests that *Skap2KO* BM neutrophils are defective for invasin-β1-mediated phagocytosis regardless of the presence of YopH or the T3SS. As expected, a significantly higher percentage of *ΔyscF* was phagocytosed in WT BM neutrophils than the WT-*Yptb* and *ΔyopH* mutant (Fig 7). The *ΔyopH* mutant was consistently phagocytosed by WT BM neutrophils more frequently than WT-*Yptb*, but this difference was not statistically significant suggesting that YopH has a modest role in preventing phagocytosis in WT BM neutrophils.

In summary, YopH inhibits ROS production, reduces degranulation and has a modest effect on phagocytosis of WT-*Yptb*. *Skap2KO* neutrophils have defects in ROS production and phagocytosis, but retain the ability to migrate to sites of *Yptb* infection and degranulate. Collectively, these results establish a role for SKAP2-mediated neutrophil signaling and its antimicrobial function towards *Yptb* and reveal how YopH acts to counter it.

## Discussion

By exploring the interplay between *Yptb* effector YopH and the neutrophil adaptor protein, SKAP2, during tissue infection, we demonstrate the role YopH plays in protecting *Yptb* from neutrophil-mediated ROS production as well as the role SKAP2 plays in mediating neutrophil antimicrobial functions. The finding that inactivation of SKAP2 partially restores the growth of a Δ*yopH* mutant indicates that one or more SKAP2-controlled pathways must be inactivated by YopH for full virulence of *Yptb*. Thus, among the various proteins that have been previously identified as targets of YopH in neutrophils, macrophages, epithelial cells and T cells [29, 36, 44, 81, 83–87], SKAP2-controlled functions in neutrophils play a non-redundant role in host immune defenses in controlling *Yptb* infection. To our knowledge, this is the first identified signal-transduction pathway essential for YopH to target during *Yptb* infection in mammalian tissues.

**Fig 8:**
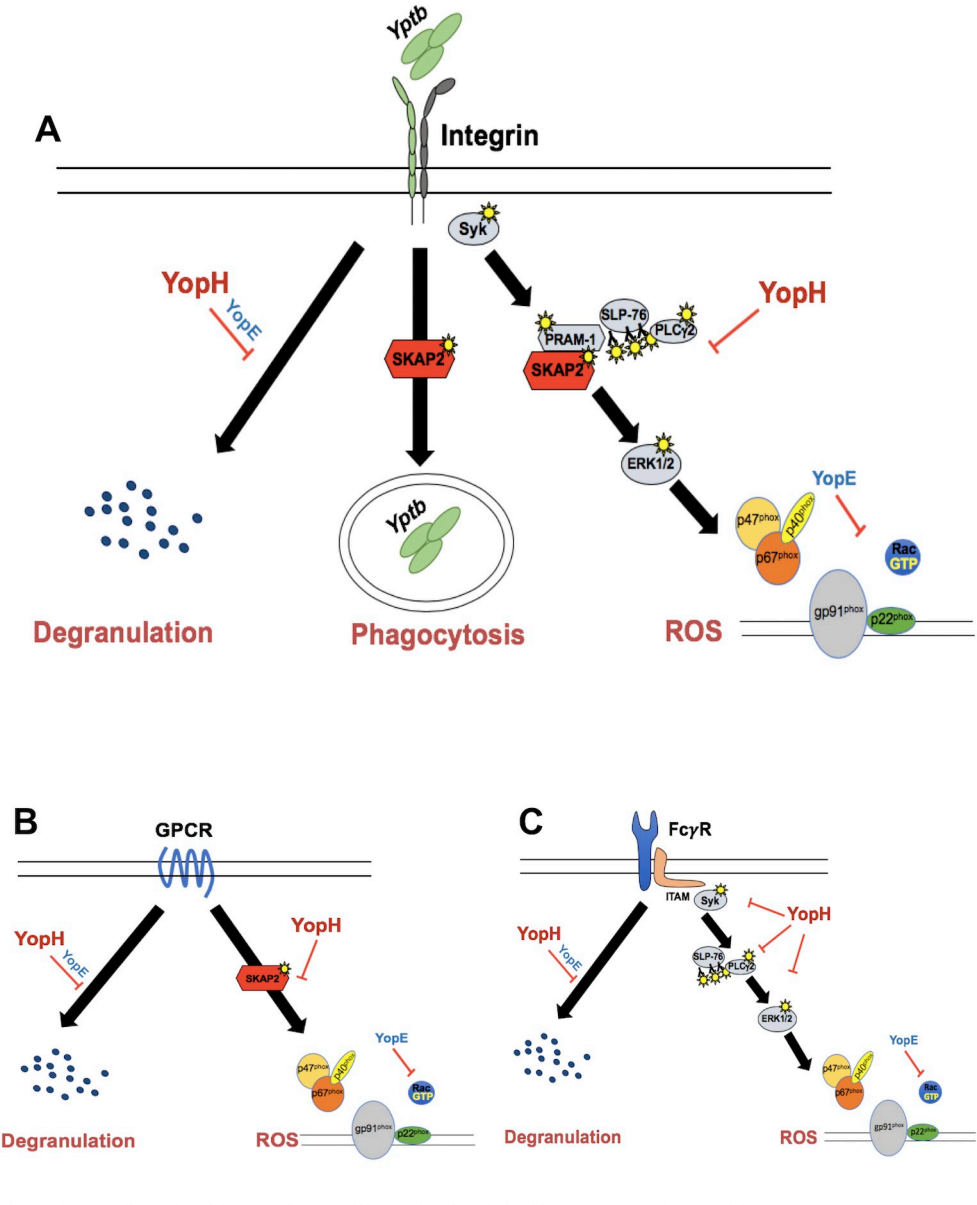
YopH dephosphorylates SKAP2-dependent and independent signaling proteins inhibiting ROS production and degranulation in neutrophils. (A) After integrin engagement, YopH inhibits signal propagation that is required for the activation of the NADPH oxidase complex and degranulation while SKAP2 is essential for ROS and phagocytosis and for maximal ERK1/2 phosphorylation, but not for degranulation or phosphorylation of Syk, SLP-76 and PLCγ2. (B) After GPCR stimulation of LPS-primed neutrophils with fMLP, YopH inhibits ROS and degranulation, where SKAP2 is required for ROS production, but not degranulation. (C) YopH inhibits ROS and degranulation after FcγR stimulation and reduces phosphorylation of Syk, SLP-76, PLCγ2 and ERK1/2, but SKAP2 is not required for ROS production, degranulation, nor phosphorylation of these signaling proteins.

What is YopH doing during infection? Our results indicate that YopH plays at least two important roles in blocking antimicrobial functions of neutrophils: inhibiting ROS production and reducing degranulation (Fig 8). A Δ*yopH* mutant survived significantly better in *gp91*^*phox-/-*^ mice than in WT mice, and YopH fully blocked ROS production downstream of integrin receptor, GPCR and FcγR stimulation in neutrophils. Furthermore, YopH interfered with the phosphorylation cascade triggered downstream of FcγR stimulation, including phosphorylation of Syk, SLP-76, PLCγ2 and ERK1/2. Syk was not identified as a YopH target in a previous study; however, in that case, neutrophils were activated with bacteria alone [36] raising the possibility that Syk may be inactivated by YopH *in vivo* if it is activated through concurrent stimulation of several receptors at infectious loci. During tissue infection, other SKAP2-independent pathways may be triggered to generate ROS, which YopH can block, thus a Δ*yopH* mutant is susceptible to the ROS produced. In addition, in some cases NETosis depends on ROS production [65]. So our findings that YopH is critical to block ROS production support the idea that YopH may also block NETosis observed after infection of human neutrophils with a TT3S-minus strain but not WT *Yptb* strain [88]. We also found that degranulation was not dependent on SKAP2 after integrin and GPCR activation, receptors triggered by *Yersinia* [18, 19, 22–25]. Thus, degranulation could contribute to the partial elimination of the *ΔyopH* mutant observed in tissue infection of the *Skap2KO* mice (Fig 2A), because YopH was sufficient to reduce MMP-9 release from all three receptors tested (Fig 6A–6C). In addition, others have shown that YopH and YopE block degranulation-dependent release of lactoferrin, a molecule in secondary granules [40, 41]. Interestingly, infecting neutrophils with a *Δ5* mutant induced a significantly higher MMP-9 release than the activation of a single receptor with its corresponding ligand (S6B Fig). These results suggest concurrent stimulation of several receptors during infection can occur, which results in activation of multiple signaling pathways to effectively magnify the response. This may occur in a tissue infection milieu where neutrophils are subjected to numerous innate signals, such as integrin stimulation from *Yptb* binding, and LPS and fMLP shed from *Yptb*. While it is tempting to speculate that YopH prevents phagocytosis in tissue infection as has been observed in isolated phagocytes [39], internalized Δ*yopH* was recovered in infected tissues at levels comparable to WT-*Yptb* [36], suggesting that YopH does not contribute significantly to anti-phagocytosis activities of *Yptb* in this model of *Yptb* tissue infection.

Precedence for more than one Yop blocking similar antimicrobial functions by targeting different proteins in receptor-mediated signaling cascades is abundant [5] and suggests that *Yptb* uses its arsenal of 6–7 Yops to block antimicrobial functions triggered by activation of different receptors and/or to alter antimicrobial responses of different cellular microdomains. Here, YopH was both necessary and sufficient to block ROS production downstream of two receptor classes; integrin and FcγR, however, its absence only partially crippled GPCR-mediated ROS production after infection with a Δ*yopH* mutant. These finding are consistent with the essential role of YopH in blocking integrin-mediated calcium flux, but not GPCR-mediated calcium release [38], and show that other Yops can compensate after activation of certain receptors. One Yop that could be acting with YopH to block GPCR-mediated ROS is YopO, as it has been reported to target Rho, Rac, and Gαq-dependent signaling pathways of GPCR [89–95]. In contrast to the near complete restoration of growth of a Δ*yopH* mutant in *gp91*^*phox-/-*^ mice reported here, a Δ*yopE* mutant was not restored for growth although a chimeric YopE that retained its Rac1 GAP but was deficient in other GAP activities was significantly restored for growth in *gp91*^*phox-/-*^ mice compared to C57BL/6J mice [67]. Thus, YopH appears to play a major role in protecting *Yptb* from ROS production during tissue infection, in part through its ability to block SKAP2-controlled pathways, while YopE may play a more prominent role at the phagocytic cup in inactivating Rac1 and RhoG rather than in inactivating Rac2 [67, 96–100].

Neutrophils kill bacteria using several different mechanisms including phagocytosis, ROS production, degranulation and NET production [101–103]. This raised the question of what anti-microbial functions are controlled by SKAP2, as *Skap2KO* mice partially restored Δ*yopH* growth. Together, our findings uncouple several key integrin- and GPCR-dependent antimicrobial responses from FcγR-dependent responses by virtue of their SKAP2-dependency for ROS production, and show that this adaptor is critical for some, but not all integrin- and GPCR-driven antimicrobial functions in neutrophils (Fig 8). SKAP2 may be part of a phospho-relay and/or could act a scaffolding function needed for full ERK1/2 phosphorylation [49]. ERK1/2 is required for phosphorylation of the p47^phox^ and p67^phox^ subunits of the NADPH oxidase complex in response to various stimuli in human neutrophils and therefore for ROS production [76–79]. The scaffolding function of SKAP2 may be essential to form stable signaling complexes downstream of integrin receptor and/or GPCR, but not FcγR. Hence, the defect of ROS in the integrin pathway may not be due to alteration of the phosphorylation state or activity of the proximal signaling proteins, but rather due to displacement of these proteins from the plasma membrane in the absence of SKAP2. In line with this hypothesis, the membrane localization of the SKAP2 binding partner, WASp, but not its phosphorylation, is abolished in *Skap2KO* neutrophils. This suggests that phosphorylation alone is not the only critical mediator of its activity, and possibly other SKAP2-associated-protein activities [49].

The fact that SKAP2 is critical for GPCR mediated ROS production was surprising, as the majority of the proteins known to interact with or predicted to be in the same signaling pathway as SKAP2 in neutrophils and other immune cells are dispensable downstream of GPCR; these include Syk, SLP-76, PRAM-1, and PLCγ2 [51, 53, 54, 104], suggesting that SKAP2 signals through a different signaling pathway downstream of GPCR (Fig 8B). In addition, SKAP2 is not required for calcium flux in neutrophils following chemokine stimulation [49]. However, previous studies employed cytochalasin B to amplify the fMLP signal [51, 53, 54, 104], rather than LPS to prime neutrophils. Therefore, SKAP2 could be required for signaling pathways downstream of TLR4 receptor and/or downstream of GPCR in ROS production. Consistent with these findings, the proliferative response to LPS in *Skap2KO* B cells is strongly impaired, highlighting the critical role of SKAP2 in TLR4-mediated activation [70]. On the other hand, LPS-induced cytokine production was normal in *Skap2KO* dendritic cells, supporting the different functional requirement of SKAP2 in LPS activation in different immune cell types and functions [105]. Further, it is critical to evaluate these pathways as well as the role of SKAP2 in antimicrobial functions in human neutrophils to determine if these findings are applicable to humans as mouse and human neutrophils differ [55, 75, 103, 106].

While SKAP2 is necessary for limiting colonization of Δ*yopH* mutant, it does not appear to limit neutrophil migration to spleens in our infection models. This contrasts with the defect observed in a sterile inflammation model of hepatic necrosis where *Skap2KO* neutrophils were defective for migration [49]. It is noteworthy that the time courses of the two studies were different. In the sterile inflammation model, neutrophil recruitment was first observed after 30 min of tissue injury and infiltration reached maximum by 4 hours, whereas in our infection model no migration was observed for the first 24 hours in the spleen. Consistent with our results, neutrophils lacking SLP-76, which functions downstream of integrin receptors, also showed normal migration *in vivo* in response to *Staphylococcus aureus* infection, yet were defective in a sterile inflammation model of acute kidney injury [51, 107]. The difference in SKAP2-dependency in the context of microbial infection versus sterile inflammation may be due to multiple factors, including different chemokines released upon infection, a different adaptor protein compensating for the absence of SKAP2 in the infection model, or additional SKAP2-independent receptors activated during infection for tissue extravasation [51, 107].

In conclusion, *Yptb* employs YopH to target both SKAP2-dependent and independent signal-transduction pathways to cripple antimicrobial functions of murine neutrophils. *Skap2KO* neutrophils migrate efficiently to infected tissues, but only partially control the growth of a *ΔyopH* mutant. SKAP2 is required for ROS production after integrin and GPCR stimulation but not for degranulation, indicating that different antimicrobial functions can be triggered by activation of the same receptor but are not all dependent on SKAP2. However, since all these activities are blocked by YopH, future work is focused on uncovering the additional signal-transduction pathways that are essential YopH targets during tissue infection.

## Materials and methods

### Bacterial strains and growth conditions

Strains used in this work are listed in S1 Table. All mouse infections used *Yptb* IP2666 or derivatives while some neutrophil assays were performed with *Yptb* YPIIIpIB1 and derivatives as described in Figure Legends. The kanamycin resistant (Kan^R^) IP2666*ΔyopH* mutant was constructed by introducing a *yopH* deletion into IP2666-NdeI-Kan^R^ as previously described [30]. *Yptb* were grown overnight at 26°C in 2xYT media with aeration. For animal infections, overnight cultures were diluted 1:40 in 2xYT media incubated at 26°C with aeration for 8 hours, after which they were diluted 1:100 and incubated overnight with aeration and the OD_600_ measured. The bacteria were diluted in PBS to achieve the required input dose for intravenous (I.V.) or intratracheal (I.T.) inoculation. For infection of neutrophils, *Yptb* were grown under Yop-inducing conditions as described [36]. Strains containing pBAD plasmids were grown in presence of 20 μg/ml chloramphenicol and 50 mM arabinose was added to the culture prior to the shift to 37°C and maintained in medium with antibiotics and inducer throughout the assays. OD_600_ of each culture was measured and strains were diluted in KRP buffer (Krebs-Ringer-Phosphate: PBS—Corning; Cat# 21040CV + 1 mM CaCl_2_ + 1.5 mM MgCl_2_) with 5 mM glucose or 5 mM arabinose depending on bacterial strains used in infections. BM neutrophils were infected at the indicated MOI.

### Mouse infections

Female 8–12 week old BALB/c (Taconic labs), BALB/c-*Skap2KO* [48], C57BL/6J (Jackson labs), and C57BL/6J *gp91*^*phox-/-*^ (Jackson labs) mice were used for infections. Competition infections were performed using an equal mixture of unmarked and Kanamycin-resistant marked IP2666 strains. C57BL/6J and *gp91*^*phox-/-*^ were I.V. or I.T. infected with 1x10^3^ or 5x10^3^ colony forming units (CFU), respectively, with a 1:1 mixture of WT-*Yptb* IP2666 and *YptbΔyopH*- Kan^R^. Two days post infection spleens and lungs were isolated, weighed, homogenized and plated on L agar containing 0.5μg/mL irgasan. BALB/c and *Skap2KO* mice were inoculated I.V. with 1x10^3^ WT-*Yptb* IP2666 and Kan^R^ marked *ΔyopH* and mice were sacrificed three days post-infection. The competitive index (C.I.) (C.I. = (CFU_Δ*yopH*_/CFU_WT_)_output_/(CFU_Δ*yopH*_/CFU_WT_)_input_) was calculated as described [108] with the following modifications. In some cases, the CFU for each strain were determined by subtracting the number of *yopH*-Kan^R^ colonies from the total number of colonies recovered from L-irgasan plates to increase the limit of detection. For neutrophil (Ly6G^+^) cell depletions, mice were intraperitoneally injected with 100μl of 50μg/mL of 1A8 antibody (α-Ly6G^+^) (Fisher; Cat # bdb551459) 16 hours prior to and 24 hours post-infection. Depletion was confirmed by staining spleen homogenates with α-GR1^+^ and α-CD11b^+^ and analysis by FACS as previously described [21]. For determining the migration kinetics of neutrophils, 8 week-old female BALB/c and *Skap2KO* were either mock infected or IV infected with 1x10^3^ WT-*Yptb-*GFP strain (chloramphenicol, Cm^R^). Spleens were collected at 24, 33, 48 hours post-infection for CFUs and immunohistochemistry.

### Immunofluorescence and image analysis

Spleen tissues collected at 24, 33 and 48 hours were fixed in 4% paraformaldehyde for 4 hours at room temperature. Tissues were frozen-embedded in Sub Xero freezing media (Mercedes Medical; Cat # MER 5000) and cut into 10 μm sections using a cryostat microtome. Sections were permeabilized using 100% methanol followed by blocking with 2% BSA in PBS for 1 hour at room temperature. Ly6G^+^ cells were detected by staining sections with 1:100 rat anti-mouse monoclonal Ly6G (clone 1A8) antibody for 1 hour, washing 3 times with PBS, then incubating with 1:500 goat anti-rat Alexa Fluor 594 antibody (Invitrogen Cat# A-11007) and 1:1000 DAPI (Invitrogen) for 1 hour, and washing 3 times with PBS. Finally, samples were mounted on glass slides using ProLong Gold antifade (Invitrogen, Cat# P36930) and cured overnight in the dark at room temperature. Tissues were imaged using a 40X objective on a Nikon A1R confocal microscope (Nikon Instruments Inc.), with 0.5 μm z-steps using a filter set for DAPI (Ex/Em:350/470nm), GFP (Ex/Em:488/525nm) and mCherry (Ex/Em:561/595nm). Volocity software 6.3 was used for 3D reconstruction image quantification. For quantification of the microcolony size by volume, uninfected control images were used to set the threshold of the GFP intensity signal and objects smaller than 1μm^3^ were excluded. Volume was determined by the summation of the individual signals in the green channel after applying the threshold limits. Quantification of neutrophils was determined by determining the sum of the red fluorescent signal intensity (mCherry channel).

### Neutrophil isolation

BM neutrophils were isolated as previously described [36]. Briefly, BM was harvested from tibia and femur bones in HBSS without Ca^2+^ or Mg^2+^ (HBSS-/-). Red blood cells were lysed using sterile endotoxin free HyClone cold water (GE Heathcare; Cat# SH30529.FS) followed by addition of HBSS-/- to restore normal tonicity. Neutrophils were isolated using a three step, 55%, 65%, 75%, Percoll density gradient and centrifuged at 480 x g for 30 min without brakes. The cells at the 65–75% interface were collected, washed 2 times with HBSS-/-, and rested at room temperature for one hour. Purity of neutrophils ranged from 82–89% by FACS analysis with anti-CD11b, anti- Ly6C and anti-Ly6G antibodies (BioLegend). Neutrophils were then re-suspended in KRP neutrophil buffer to the desired concentration incubated for 20 min at room temperature and shifted to 37°C in 5% CO_2_ for 10–15 min prior to initiation of the experiment.

### Neutrophil ROS assays

For stimulating integrin receptors, wells were coated with 15μg/mL poly-RGD (Sigma; Cat# F5022) for 2 hours at 37°C, and then washed 2 times with PBS. To stimulate FcγR, wells were coated with 20μg/ml human serum albumin (Sigma; Cat # A9511) for 1 hour at 37°C, washed 2X with PBS, incubated with 10% FBS for 30 min, washed 2 times with PBS, then incubated with a 1:400 dilution of rabbit anti-human serum albumin (Sigma; Cat # A0433) in 10% FBS for 1 hour at 37°C, followed by washing 2 times with PBS. Control wells were coated with 10% FBS for 30 min, then washed 2 times with PBS. Assays for GPCR receptor were performed in the absence of Mg^2+^ to decrease the possibility of integrin dependent responses and surfaces were coated with 10% FBS to minimize adhesion to mimic cells in suspension. For stimulating GPCR, BM neutrophils were suspended in KRP without Mg^2+^ and primed with 10μg/mL LPS (Sigma; Cat# L4516) for 20 min then added to 10% FBS coated wells without centrifugation to maintain cells in suspension followed by stimulation using 1μM fMLP (Sigma; Cat # F3506). BM neutrophils suspended in KRP buffer at 37°C were loaded with 50 μM isoluminol (Sigma; Cat# A8264) and 15 U/ml of HRP (Sigma; Cat # P6782) as previously described [109] and 2 x10^5^ neutrophils/well were added to 96-well white high-bound plates (Thermo Fisher; Cat# 3922) coated with 10% FBS or different ligands and centrifuged at 190 x g for 1 min. For infections, bacteria resuspended in KRP buffer were added to wells at a MOI of 20:1 simultaneously with neutrophils when stimulating integrin and Fcγ receptors. The bacteria and neutrophils were spun down on the ligand coated plates for 1 min to uniformly induce receptor binding. For GPCR stimulation, bacteria were added to neutrophils with LPS priming and kept in suspension. PMA (1μM) (Sigma; Cat# P1585) was used as a positive control. Chemiluminescence was measured using a BioTek Synergy HT microplate reader. ROS detection using cytochrome c (Sigma; Cat # C-7752) was performed as previously described [109–110] with the following modifications. BM neutrophils (1X10^5^ per 50 μl) were added to 50 μl 200μM cytochrome c in 96 well high-binding Immulon 4HBX plates (Fisher; Cat# 3855) coated with ligands or FBS. Absorbance at 550nm and 490nm were recorded and the difference used to calculate the nanomoles of superoxide produced using an extinction coefficient of 2.11x10^4^ M^-1^cm^-1^ for cytochrome c. The total amount of ROS for each condition was corrected by subtracting its superoxidase dismutase (SOD) control [109].

### Neutrophil degranulation assay

2 x 10^5^ BM neutrophils/well were plated on 96 well Immulon 4HBX plates coated with poly-RGD, IC or 10% FBS and incubated at 37°C, 5% CO_2_. Infections were carried out at a MOI of 20:1 as described for ROS assays. After 3 hours, to determine the total amount of MMP-9, the control wells were treated with 0.01% triton X-100, without removing the supernatant, to lyse the cells and the supernatant collected. To determine the amount of released MMP-9, plates were placed on ice for 5 min to stop the reaction and then centrifuged for 10 min at 4°C at 190 x g. Supernatants were collected and stored at -80°C until analysis by ELISA using DuoSet (R&D Systems; Cat # DY6718) for mouse MMP-9, or by western blot using an antibody against Rho-GDI (Cell Signaling; Cat# 2564S). The remaining cells in the wells were collected and lysed for western blot analysis to determine the total amount of Rho-GDI using an antibody against Rho-GDI. For comparisons between WT and *Skap2KO* neutrophils the absolute amount of MMP-9 detected in supernatants was reported for the stimulated receptor after subtracting the unstimulated (FBS-coated wells) control values. The amount of MMP-9 released after infection with *Yptb* strains was reported as fold-change compared to the control of unstimulated, uninfected wells.

### Western blot analysis

Cells were incubated at 37°C, 5% CO_2_ for 5 min or 10 min for FcγR or integrin stimulation respectively. In some cases, BM neutrophils were infected with YPIII WT-*Yptb* or Δ*yopH* for 30 min and then spun down on the ligand coated surface. Cells were lysed in 1X Novex buffer and 2x10^6^ cell equivalents were resolved on a 4–12% NuPAGE gel (Invitrogen; Cat# NP0335BOX) in MOPS buffer. Proteins were transferred to Immobilon-FL filters and subjected to Western blot analysis with the following primary antibodies at a dilution of 1:500 anti-Syk (Cell Signaling; Cat# 2712), anti-SykpY352 (Cell Signaling Cat#2701), anti-SLP76 clone AS55 (Millipore; Cat# 05–1426), anti- SLP76pY128 (BD Pharmingen; Cat# 558367), anti-PLCγ2 (Cell Signaling; Cat# 3872), anti-PLCγ2pY1217 (Cell Signaling; Cat# 3871), anti-Erk1/2 pThr202/pTyr204 (Cell Signaling; Cat# 4370), anti-Erk1/2 (Cell Signaling; Cat# 9102), or at 1:1000 of anti-Rho-GDI (Cell Signaling; Cat# 2564S). Secondary LI-COR goat anti-mouse antibody IRDye 800 CW (Cat# 827–08364) and goat anti-rabbit IRDye 800 CW (Cat# 926–32211) were used at a dilution 1:20,000. ODYSSEY CLx LI-COR system was used to image the blots and IS Image Studio used for analysis and quantification of the bands.

### Gentamicin protection assay

YPIII *WT-Yptb*, Δ*yopH* or Δ*yscF* strains were grown under Yop-inducing conditions and used to infect 1 x10^4^/well BM neutrophils at MOI of 10:1 in 96 well Immulon 4HBX plates coated with 10% FBS for 30 min at 37°C, 5% CO_2_. Gentamicin was added to a final concentration of 100μg/ml for 60 min, and then cells were washed three times with PBS. BM neutrophils were centrifuged at 190 x g for 5 min between each wash. BM neutrophils were lysed with 100 μl 0.5% Triton-X-100 for 10 min followed by addition of 100μl 2xYT, and lysates were plated at different dilutions to determine the number of intracellular bacteria. Results are reported as the percent of the recovered CFU from gentamicin-treated cells divided by the non-gentamicin-treated control.

### Statistical analyses

Statistical analyses were performed in GraphPad Prism software (version 7b). Specific tests used are indicated in the figure legends. Significant difference is indicated as *p<0.05, **p <0.01, ***p<0.001, ****p<0.0001, and ns = non-significant for all figures.

### Ethics statement

This study was performed in accordance with the recommendations in the Guide for Care and Use of Laboratory Animals of the National Institutes of Health. The Tufts University Institutional Animal Care and Use Committee (IACUC) reviewed the study protocol under approval number B2015-35 and B2018-10.

## Supporting information

S1 Fig *Yptb* CFU in spleen of C57BL/6J or C57BL/6J *gp91*^*phox-/-*^ mice and assessment of bone marrow neutrophil purity. (A) C57BL/6J or C57BL/6J *gp91*^*phox-/-*^ mice were I.V. inoculated with a 1:1 mixture of IP2666 WT-*Yptb* and Δ*yopH*-Kan^R^. Spleen were collected 2 days post-infection and CFU for WT-*Yptb* and Δ*yopH*-Kan^R^ was determined by plating on selective and non-selective plates. Statistical significance was calculated using one-way ANOVA with Sidak’s multiple comparison test after log_10_ transformation of data. (B) Bone marrow was harvested from the tibia and femur bones of mice and neutrophils were isolated using Percoll density gradient method. Cells at the 65–75% interface were collected and stained using with α-CD11b, α-Ly6G and α-Ly6C and analyzed by flow cytometry. Bone marrow isolated neutrophils were more than 80% pure.
(TIFF)

S2 Fig WT *Yptb*, *ΔyopM* and Δ*5*+pYopH secrete equivalent amounts of YopH. Overnight cultures of IP2666 WT-*Yptb*, *Yptb-*Δ*5*+pYopH and *Yptb-ΔyopM* were diluted 1:40 in low calcium media and grown for 2 hours at 26°C with aeration followed by addition of 50mM arabinose to induce YopH expression from the pBAD plasmid and shifted to 37°C for 2 hours with aeration. Proteins from culture supernatants were precipitated, resolved on a 4–20% gradient polyacrylamide gel and stained using coomassie blue dye.
(TIFF)

S3 Fig BALB/c and *Skap2KO* mice have equivalent *Yptb* CFU during infection. (A) WT-BALB/c and *Skap2KO* mice were intraperitoneally injected with 1A8 or an isotype control antibody and 16 hrs later inoculated I.V. with an equal mixture of 10^3^ CFU IP2666 WT-*Yptb* and *ΔyopH-*Kan^R^. Spleens were collected 3 days post-infection and plated for CFUs. Total CFU are shown. (B)WT-BALB/c and *Skap2KO* mice were I.V. infected with 1x10^3^ CFU *Yptb*-GFP, and sacrificed at 24, 33, or 48 hours post-infection. Spleens were collected, weighed, homogenized, and plated for CFU on selective plates. Each dot represents a mouse; horizontal bars represent the geometric mean. Significance was calculated using (A) two-way ANOVA followed by Tukey’s Post-test and (B) Student’s t-test.
(TIFF)

S4 Fig *Skap2KO* BM neutrophils fail to produce ROS following integrin and GPCR receptor stimulation but not after FcγR stimulation. Respiratory burst of WT-BALB/c and *Skap2KO* BM neutrophils (1x10^5^ cells) was measured using the cytochrome c reduction test by (A-B) plating on a poly-RGD surface, (C-D) priming with 10μg/ml LPS for 20 min followed by stimulation with 1μM fMLP, or (E-F) plating on an IC coated surface. Absorbance at 550 nm and 490 nm was recorded and each condition was corrected by its superoxidase dismutase (SOD) control value. Total amount of ROS was estimated from measuring the AUC for each condition for the duration of the experiment. Data are shown as the means ± SD from triplicate measurements of one experiment, which are representative of at least 4 independent experiments. Statistical significance was calculated using Student t-test.
(TIFF)

S5 Fig *Skap2KO* mice are defective for SKAP2 and do not overexpress SKAP1. Lysates of BM neutrophils isolated from WT-BALB/c and *Skap2KO* were immunoblotted for SKAP2 and SKAP1. Anti-Rho-GDI was used as a loading control. No band for SKAP2 was detected in *Skap2KO* neutrophils confirming the absence of SKAP2. SKAP1 was detected at very low levels in both genotypes.
(TIFF)

S6 Fig Release of neutrophil granules is not due to cell lysis and degranulation induced after infection and receptor stimulation is significantly higher than stimulation of single receptors alone. (A) Supernatants and cell lysates (2 x 10^5^ cells) from infected and stimulated samples were immunoblotted with anti-Rho-GDI as an indicator of cell lysis. A positive control for degranulation was treatment with PMA, and the negative control was cells added to FBS-coated wells. Rho-GDI was detected in the supernatants of cells treated with triton X-100. (B) BM neutrophils (2x10^5^) were stimulated with poly-RGD, fMLP or IC to stimulate integrin, GPCR, or FcγR respectively and left uninfected or simultaneously infected with *Yptb*Δ5 at a MOI of 20:1 for 3 hrs. Cell free supernatants from equivalent numbers were analyzed by ELISA for MMP-9 release. Data presented are expressed as fold increase relative to control uninfected and unstimulated samples. The data represent the means ± SEM from 3 independent experiments done in 2–3 replicates. (C) WT-BALB/c and *Skap2KO* BM neutrophils were stimulated for 3 hrs by 1μM PMA. Cell-free supernatants were analyzed for MMP-9 by ELISA. The data represent the means ± SEM from 3 independent experiments done in triplicate. Statistical significance was calculated using Student t-test.
(TIFF)

S1 Table List of strains. (DOCX)

## References

[1] Viboud GI, Bliska JB. Yersinia outer proteins: role in modulation of host cell signaling responses and pathogenesis. Annual review of microbiology. 2005;59:69–89. 10.1146/annurev.micro.59.030804.121320 . 15847602

[2] McNally A, Thomson NR, Reuter S, Wren BW. 'Add, stir and reduce': Yersinia spp. as model bacteria for pathogen evolution. Nat Rev Microbiol. 2016;14(3):177–90. Epub 2016/02/16. 10.1038/nrmicro.2015.29 . 26876035

[3] Wren BW. The yersiniae—a model genus to study the rapid evolution of bacterial pathogens. Nat Rev Microbiol. 2003;1(1):55–64. 10.1038/nrmicro730 . 15040180

[4] Cornelis GR. The type III secretion injectisome. Nat Rev Microbiol. 2006;4(11):811–25. Epub 2006/10/17. 10.1038/nrmicro1526 . 17041629

[5] Bliska JB, Wang X, Viboud GI, Brodsky IE. Modulation of innate immune responses by Yersinia type III secretion system translocators and effectors. Cell Microbiol. 2013;15(10):1622–31. Epub 2013/07/10. 10.1111/cmi.12164 23834311 PMC3788085

[6] Coburn B, Sekirov I, Finlay BB. Type III secretion systems and disease. Clin Microbiol Rev. 2007;20(4):535–49. Epub 2007/10/16. 10.1128/CMR.00013-07 17934073 PMC2176049

[7] Green ER, Mecsas J. Bacterial Secretion Systems: An Overview. Microbiol Spectr. 2016;4(1). Epub 2016/03/22. 10.1128/microbiolspec.VMBF-0012-2015 26999395 PMC4804464

[8] Loeven NA, Medici NP, Bliska JB. The pyrin inflammasome in host-microbe interactions. Curr Opin Microbiol. 2020;54:77–86. Epub 2020/03/03. 10.1016/j.mib.2020.01.005 . 32120337 PMC7247927

[9] Peterson LW, Brodsky IE. To catch a thief: regulated RIPK1 post-translational modifications as a fail-safe system to detect and overcome pathogen subversion of immune signaling. Curr Opin Microbiol. 2020;54:111–8. Epub 2020/02/25. 10.1016/j.mib.2020.01.015 . 32092691

[10] Sanchez-Garrido J, Slater SL, Clements A, Shenoy AR, Frankel G. Vying for the control of inflammasomes: The cytosolic frontier of enteric bacterial pathogen-host interactions. Cell Microbiol. 2020;22(4):e13184 Epub 2020/03/19. 10.1111/cmi.13184 . 32185892 PMC7154749

[11] Cornelis GR. Yersinia type III secretion: send in the effectors. J Cell Biol. 2002;158(3):401–8. Epub 2002/08/07. 10.1083/jcb.200205077 12163464 PMC2173816

[12] Davis KM, Mohammadi S, Isberg RR. Community behavior and spatial regulation within a bacterial microcolony in deep tissue sites serves to protect against host attack. Cell Host Microbe. 2015;17(1):21–31. Epub 2014/12/17. 10.1016/j.chom.2014.11.008 25500192 PMC4669952

[13] Crimmins GT, Mohammadi S, Green ER, Bergman MA, Isberg RR, Mecsas J. Identification of MrtAB, an ABC transporter specifically required for Yersinia pseudotuberculosis to colonize the mesenteric lymph nodes. PLoS Pathog. 2012;8(8):e1002828 Epub 2012/08/10. 10.1371/journal.ppat.1002828 22876175 PMC3410872

[14] Balada-Llasat JM, Mecsas J. Yersinia has a tropism for B and T cell zones of lymph nodes that is independent of the type III secretion system. PLoS Pathog. 2006;2(9):e86 Epub 2006/09/05. 10.1371/journal.ppat.0020086 16948531 PMC1557584

[15] Durand EA, Maldonado-Arocho FJ, Castillo C, Walsh RL, Mecsas J. The presence of professional phagocytes dictates the number of host cells targeted for Yop translocation during infection. Cell Microbiol. 2010;12(8):1064–82. 10.1111/j.1462-5822.2010.01451.x 20148898 PMC2906667

[16] Marketon MM, DePaolo RW, DeBord KL, Jabri B, Schneewind O. Plague bacteria target immune cells during infection. Science. 2005;309(5741):1739–41. Epub 2005/07/30. 10.1126/science.1114580 16051750 PMC3210820

[17] Koberle M, Klein-Gunther A, Schutz M, Fritz M, Berchtold S, Tolosa E, et al Yersinia enterocolitica targets cells of the innate and adaptive immune system by injection of Yops in a mouse infection model. PLoS Pathog. 2009;5(8):e1000551 Epub 2009/08/15. 10.1371/journal.ppat.1000551 19680448 PMC2718809

[18] Isberg RR, Leong JM. Multiple beta 1 chain integrins are receptors for invasin, a protein that promotes bacterial penetration into mammalian cells. Cell. 1990;60(5):861–71. 10.1016/0092-8674(90)90099-z . 2311122

[19] Isberg RR, Barnes P. Subversion of integrins by enteropathogenic Yersinia. J Cell Sci. 2001;114(Pt 1):21–8. Epub 2000/12/12. . 11112686 10.1242/jcs.114.1.21

[20] Maldonado-Arocho FJ, Green C, Fisher ML, Paczosa MK, Mecsas J. Adhesins and host serum factors drive Yop translocation by yersinia into professional phagocytes during animal infection. PLoS Pathog. 2013;9(6):e1003415 Epub 2013/07/03. 10.1371/journal.ppat.1003415 23818844 PMC3688556

[21] Paczosa MK, Fisher ML, Maldonado-Arocho FJ, Mecsas J. Yersinia pseudotuberculosis uses Ail and YadA to circumvent neutrophils by directing Yop translocation during lung infection. Cell Microbiol. 2014;16(2):247–68. 10.1111/cmi.12219 24119087 PMC3981955

[22] Hoffmann R, van Erp K, Trulzsch K, Heesemann J. Transcriptional responses of murine macrophages to infection with Yersinia enterocolitica. Cell Microbiol. 2004;6(4):377–90. Epub 2004/03/11. 10.1111/j.1462-5822.2004.00365.x . 15009029

[23] Handley SA, Dube PH, Miller VL. Histamine signaling through the H(2) receptor in the Peyer's patch is important for controlling Yersinia enterocolitica infection. Proceedings of the National Academy of Sciences of the United States of America. 2006;103(24):9268–73. Epub 2006/05/24. 10.1073/pnas.0510414103 16717182 PMC1482599

[24] Osei-Owusu P, Charlton TM, Kim HK, Missiakas D, Schneewind O. FPR1 is the plague receptor on host immune cells. Nature. 2019;574(7776):57–62. Epub 2019/09/20. 10.1038/s41586-019-1570-z . 31534221 PMC6776691

[25] Sheahan KL, Isberg RR. Identification of mammalian proteins that collaborate with type III secretion system function: involvement of a chemokine receptor in supporting translocon activity. MBio. 2015;6(1):e02023–14. Epub 2015/02/19. 10.1128/mBio.02023-14 25691588 PMC4337563

[26] Guan KL, Dixon JE. Protein tyrosine phosphatase activity of an essential virulence determinant in Yersinia. Science. 1990;249(4968):553–6. Epub 1990/08/03. 10.1126/science.2166336 . 2166336

[27] Zhang ZY, Clemens JC, Schubert HL, Stuckey JA, Fischer MW, Hume DM, et al Expression, purification, and physicochemical characterization of a recombinant Yersinia protein tyrosine phosphatase. J Biol Chem. 1992;267(33):23759–66. Epub 1992/11/25. . 1429715

[28] Bliska JB, Guan KL, Dixon JE, Falkow S. Tyrosine phosphate hydrolysis of host proteins by an essential Yersinia virulence determinant. Proceedings of the National Academy of Sciences of the United States of America. 1991;88(4):1187–91. 10.1073/pnas.88.4.1187 1705028 PMC50982

[29] Andersson K, Carballeira N, Magnusson KE, Persson C, Stendahl O, Wolf-Watz H, et al YopH of Yersinia pseudotuberculosis interrupts early phosphotyrosine signalling associated with phagocytosis. Molecular microbiology. 1996;20(5):1057–69. 10.1111/j.1365-2958.1996.tb02546.x . 8809758

[30] Logsdon LK, Mecsas J. Requirement of the Yersinia pseudotuberculosis effectors YopH and YopE in colonization and persistence in intestinal and lymph tissues. Infection and immunity. 2003;71(8):4595–607. 10.1128/IAI.71.8.4595-4607.2003 12874339 PMC166012

[31] Trulzsch K, Sporleder T, Igwe EI, Russmann H, Heesemann J. Contribution of the major secreted yops of Yersinia enterocolitica O:8 to pathogenicity in the mouse infection model. Infection and immunity. 2004;72(9):5227–34. Epub 2004/08/24. 10.1128/IAI.72.9.5227-5234.2004 15322017 PMC517446

[32] Fisher ML, Castillo C, Mecsas J. Intranasal inoculation of mice with Yersinia pseudotuberculosis causes a lethal lung infection that is dependent on Yersinia outer proteins and PhoP. Infection and immunity. 2007;75(1):429–42. Epub 2006/11/01. 10.1128/IAI.01287-06 17074849 PMC1828392

[33] Logsdon LK, Mecsas J. The proinflammatory response induced by wild-type Yersinia pseudotuberculosis infection inhibits survival of yop mutants in the gastrointestinal tract and Peyer's patches. Infection and immunity. 2006;74(3):1516–27. Epub 2006/02/24. 10.1128/IAI.74.3.1516-1527.2006 16495522 PMC1418670

[34] Di Genaro MS, Waidmann M, Kramer U, Hitziger N, Bohn E, Autenrieth IB. Attenuated Yersinia enterocolitica mutant strains exhibit differential virulence in cytokine-deficient mice: implications for the development of novel live carrier vaccines. Infection and immunity. 2003;71(4):1804–12. Epub 2003/03/26. 10.1128/IAI.71.4.1804-1812.2003 12654794 PMC152075

[35] Dave MN, Silva JE, Elicabe RJ, Jerez MB, Filippa VP, Gorlino CV, et al Yersinia enterocolitica YopH-Deficient Strain Activates Neutrophil Recruitment to Peyer's Patches and Promotes Clearance of the Virulent Strain. Infection and immunity. 2016;84(11):3172–81. Epub 2016/08/24. 10.1128/IAI.00568-16 27550935 PMC5067750

[36] Rolan HG, Durand EA, Mecsas J. Identifying Yersinia YopH-targeted signal transduction pathways that impair neutrophil responses during in vivo murine infection. Cell Host Microbe. 2013;14(3):306–17. 10.1016/j.chom.2013.08.013 24034616 PMC3789382

[37] Ruckdeschel K, Roggenkamp A, Schubert S, Heesemann J. Differential contribution of Yersinia enterocolitica virulence factors to evasion of microbicidal action of neutrophils. Infection and immunity. 1996;64(3):724–33. 8641773 10.1128/iai.64.3.724-733.1996 PMC173829

[38] Andersson K, Magnusson KE, Majeed M, Stendahl O, Fallman M. Yersinia pseudotuberculosis-induced calcium signaling in neutrophils is blocked by the virulence effector YopH. Infection and immunity. 1999;67(5):2567–74. 10225922 10.1128/iai.67.5.2567-2574.1999 PMC116005

[39] Grosdent N, Maridonneau-Parini I, Sory MP, Cornelis GR. Role of Yops and adhesins in resistance of Yersinia enterocolitica to phagocytosis. Infection and immunity. 2002;70(8):4165–76. Epub 2002/07/16. 10.1128/IAI.70.8.4165-4176.2002 12117925 PMC128122

[40] Taheri N, Fahlgren A, Fallman M. Yersinia pseudotuberculosis Blocks Neutrophil Degranulation. Infection and immunity. 2016;84(12):3369–78. Epub 2016/09/14. 10.1128/IAI.00760-16 27620724 PMC5116715

[41] Eichelberger KR, Jones GS, Goldman WE. Inhibition of Neutrophil Primary Granule Release during Yersinia pestis Pulmonary Infection. mBio. 2019;10(6). Epub 2019/12/12. 10.1128/mBio.02759-19 31822588 PMC6904878

[42] Pulsifer AR, Vashishta A, Reeves SA, Wolfe JK, Palace SG, Proulx MK, et al Redundant and Cooperative Roles for Yersinia pestis Yop Effectors in the Inhibition of Human Neutrophil Exocytic Responses Revealed by Gain-of-Function Approach. Infection and immunity. 2020;88(3). Epub 2019/12/25. 10.1128/IAI.00909-19 31871100 PMC7035916

[43] Moog-Lutz C, Peterson EJ, Lutz PG, Eliason S, Cave-Riant F, Singer A, et al PRAM-1 is a novel adaptor protein regulated by retinoic acid (RA) and promyelocytic leukemia (PML)-RA receptor alpha in acute promyelocytic leukemia cells. J Biol Chem. 2001;276(25):22375–81. 10.1074/jbc.M011683200 . 11301322

[44] Black DS, Marie-Cardine A, Schraven B, Bliska JB. The Yersinia tyrosine phosphatase YopH targets a novel adhesion-regulated signalling complex in macrophages. Cell Microbiol. 2000;2(5):401–14. Epub 2001/02/24. 10.1046/j.1462-5822.2000.00061.x . 11207596

[45] Marie-Cardine A, Hendricks-Taylor LR, Boerth NJ, Zhao H, Schraven B, Koretzky GA. Molecular interaction between the Fyn-associated protein SKAP55 and the SLP-76-associated phosphoprotein SLAP-130. J Biol Chem. 1998;273(40):25789–95. Epub 1998/09/25. 10.1074/jbc.273.40.25789 . 9748251

[46] Marie-Cardine A, Verhagen AM, Eckerskorn C, Schraven B. SKAP-HOM, a novel adaptor protein homologous to the FYN-associated protein SKAP55. FEBS Lett. 1998;435(1):55–60. Epub 1998/10/02. 10.1016/s0014-5793(98)01040-0 . 9755858

[47] Togni M, et al Regulation of In Vitro and In Vivo Immune Functions by the Cytosolic Adaptor Protein SKAP-HOM. Molecular and cellular biology. 2005;25(18). 10.1128/MCB.25.18.8052-8063.2005 PMC1234325 16135797

[48] Alenghat FJ, Baca QJ, Rubin NT, Pao LI, Matozaki T, Lowell CA, et al Macrophages require Skap2 and Sirpalpha for integrin-stimulated cytoskeletal rearrangement. J Cell Sci. 2012;125(Pt 22):5535–45. Epub 2012/09/15. 10.1242/jcs.111260 22976304 PMC3561861

[49] Boras M, Volmering S, Bokemeyer A, Rossaint J, Block H, Bardel B, et al Skap2 is required for beta2 integrin-mediated neutrophil recruitment and functions. The Journal of experimental medicine. 2017;214(3):851–74. 10.1084/jem.20160647 28183734 PMC5339670

[50] Jordan MS, Koretzky GA. Coordination of receptor signaling in multiple hematopoietic cell lineages by the adaptor protein SLP-76. Cold Spring Harbor perspectives in biology. 2010;2(4):a002501 10.1101/cshperspect.a002501 20452948 PMC2845197

[51] Clemens RA, Newbrough SA, Chung EY, Gheith S, Singer AL, Koretzky GA, et al PRAM-1 is required for optimal integrin-dependent neutrophil function. Molecular and cellular biology. 2004;24(24):10923–32. 10.1128/MCB.24.24.10923-10932.2004 15572693 PMC533979

[52] Clemens RA, Lenox LE, Kambayashi T, Bezman N, Maltzman JS, Nichols KE, et al Loss of SLP-76 expression within myeloid cells confers resistance to neutrophil-mediated tissue damage while maintaining effective bacterial killing. J Immunol. 2007;178(7):4606–14. Epub 2007/03/21. 10.4049/jimmunol.178.7.4606 . 17372019

[53] Jakus Z, Simon E, Frommhold D, Sperandio M, Mocsai A. Critical role of phospholipase Cgamma2 in integrin and Fc receptor-mediated neutrophil functions and the effector phase of autoimmune arthritis. The Journal of experimental medicine. 2009;206(3):577–93. 10.1084/jem.20081859 19273622 PMC2699137

[54] Newbrough SA, Mocsai A, Clemens RA, Wu JN, Silverman MA, Singer AL, et al SLP-76 regulates Fcgamma receptor and integrin signaling in neutrophils. Immunity. 2003;19(5):761–9. 10.1016/s1074-7613(03)00305-4 . 14614862

[55] Nguyen GT, Green ER, Mecsas J. Neutrophils to the ROScue: Mechanisms of NADPH Oxidase Activation and Bacterial Resistance. Front Cell Infect Microbiol. 2017;7:373 Epub 2017/09/12. 10.3389/fcimb.2017.00373 28890882 PMC5574878

[56] Pollock JD, Williams DA, Gifford MA, Li LL, Du X, Fisherman J, et al Mouse model of X-linked chronic granulomatous disease, an inherited defect in phagocyte superoxide production. Nat Genet. 1995;9(2):202–9. Epub 1995/02/01. 10.1038/ng0295-202 . 7719350

[57] Paiva CN, Bozza MT. Are reactive oxygen species always detrimental to pathogens? Antioxid Redox Signal. 2014;20(6):1000–37. Epub 2013/09/03. 10.1089/ars.2013.5447 23992156 PMC3924804

[58] Dupre-Crochet S, Erard M, Nubetae O. ROS production in phagocytes: why, when, and where? J Leukoc Biol. 2013;94(4):657–70. Epub 2013/04/24. 10.1189/jlb.1012544 . 23610146

[59] Nathan C. Neutrophils and immunity: challenges and opportunities. Nat Rev Immunol. 2006;6(3):173–82. Epub 2006/02/25. 10.1038/nri1785 . 16498448

[60] Mitra S, Abraham E. Participation of superoxide in neutrophil activation and cytokine production. Biochim Biophys Acta. 2006;1762(8):732–41. Epub 2006/08/22. 10.1016/j.bbadis.2006.06.011 . 16919916

[61] Fialkow L, Wang Y, Downey GP. Reactive oxygen and nitrogen species as signaling molecules regulating neutrophil function. Free Radic Biol Med. 2007;42(2):153–64. Epub 2006/12/27. 10.1016/j.freeradbiomed.2006.09.030 . 17189821

[62] Harbort CJ, Soeiro-Pereira PV, von Bernuth H, Kaindl AM, Costa-Carvalho BT, Condino-Neto A, et al Neutrophil oxidative burst activates ATM to regulate cytokine production and apoptosis. Blood. 2015;126(26):2842–51. 10.1182/blood-2015-05-645424 26491069 PMC4692144

[63] Brinkmann V, Reichard U, Goosmann C, Fauler B, Uhlemann Y, Weiss DS, et al Neutrophil extracellular traps kill bacteria. Science. 2004;303(5663):1532–5. 10.1126/science.1092385 . 15001782

[64] Keshari RS, Verma A, Barthwal MK, Dikshit M. Reactive oxygen species-induced activation of ERK and p38 MAPK mediates PMA-induced NETs release from human neutrophils. J Cell Biochem. 2013;114(3):532–40. Epub 2012/09/11. 10.1002/jcb.24391 . 22961925

[65] Delgado-Rizo V, Martinez-Guzman MA, Iniguez-Gutierrez L, Garcia-Orozco A, Alvarado-Navarro A, Fafutis-Morris M. Neutrophil Extracellular Traps and Its Implications in Inflammation: An Overview. Front Immunol. 2017;8:81 Epub 2017/02/22. 10.3389/fimmu.2017.00081 28220120 PMC5292617

[66] Reeves EP, Lu H, Jacobs HL, Messina CG, Bolsover S, Gabella G, et al Killing activity of neutrophils is mediated through activation of proteases by K+ flux. Nature. 2002;416(6878):291–7. 10.1038/416291a . 11907569

[67] Songsungthong W, Higgins MC, Rolan HG, Murphy JL, Mecsas J. ROS-inhibitory activity of YopE is required for full virulence of Yersinia in mice. Cell Microbiol. 2010;12(7):988–1001. 10.1111/j.1462-5822.2010.01448.x 20148901 PMC2897941

[68] Isberg RR. Discrimination between intracellular uptake and surface adhesion of bacterial pathogens. Science. 1991;252(5008):934–8. Epub 1991/05/17. 10.1126/science.1674624 . 1674624

[69] Isberg RR, Tran Van Nhieu G. Binding and internalization of microorganisms by integrin receptors. Trends Microbiol. 1994;2(1):10–4. Epub 1994/01/01. 10.1016/0966-842x(94)90338-7 . 8162429

[70] Togni M, Swanson KD, Reimann S, Kliche S, Pearce AC, Simeoni L, et al Regulation of in vitro and in vivo immune functions by the cytosolic adaptor protein SKAP-HOM. Molecular and cellular biology. 2005;25(18):8052–63. Epub 2005/09/02. 10.1128/MCB.25.18.8052-8063.2005 16135797 PMC1234325

[71] Bohmer RH, Trinkle LS, Staneck JL. Dose effects of LPS on neutrophils in a whole blood flow cytometric assay of phagocytosis and oxidative burst. Cytometry. 1992;13(5):525–31. Epub 1992/01/01. 10.1002/cyto.990130512 . 1321708

[72] Chen LY, Pan WW, Chen M, Li JD, Liu W, Chen G, et al Synergistic induction of inflammation by bacterial products lipopolysaccharide and fMLP: an important microbial pathogenic mechanism. J Immunol. 2009;182(4):2518–24. Epub 2009/02/10. 10.4049/jimmunol.0713933 . 19201908

[73] Mocsai A, Zhou M, Meng F, Tybulewicz VL, Lowell CA. Syk is required for integrin signaling in neutrophils. Immunity. 2002;16(4):547–58. Epub 2002/04/24. 10.1016/s1074-7613(02)00303-5 . 11970878

[74] Graham DB, Robertson CM, Bautista J, Mascarenhas F, Diacovo MJ, Montgrain V, et al Neutrophil-mediated oxidative burst and host defense are controlled by a Vav-PLCgamma2 signaling axis in mice. J Clin Invest. 2007;117(11):3445–52. Epub 2007/10/13. 10.1172/JCI32729 17932569 PMC2000813

[75] Futosi K, Fodor S, Mocsai A. Neutrophil cell surface receptors and their intracellular signal transduction pathways. International immunopharmacology. 2013;17(3):638–50. 10.1016/j.intimp.2013.06.034 23994464 PMC3827506

[76] Kiefer F, Brumell J, Al-Alawi N, Latour S, Cheng A, Veillette A, et al The Syk protein tyrosine kinase is essential for Fcgamma receptor signaling in macrophages and neutrophils. Molecular and cellular biology. 1998;18(7):4209–20. Epub 1998/06/25. 10.1128/mcb.18.7.4209 9632805 PMC109005

[77] El Benna J, Han J, Park JW, Schmid E, Ulevitch RJ, Babior BM. Activation of p38 in stimulated human neutrophils: phosphorylation of the oxidase component p47phox by p38 and ERK but not by JNK. Arch Biochem Biophys. 1996;334(2):395–400. Epub 1996/10/15. 10.1006/abbi.1996.0470 . 8900416

[78] Dewas C, Fay M, Gougerot-Pocidalo MA, El-Benna J. The mitogen-activated protein kinase extracellular signal-regulated kinase 1/2 pathway is involved in formyl-methionyl-leucyl-phenylalanine-induced p47phox phosphorylation in human neutrophils. J Immunol. 2000;165(9):5238–44. Epub 2000/10/25. 10.4049/jimmunol.165.9.5238 . 11046057

[79] Dang PM, Morel F, Gougerot-Pocidalo MA, El Benna J. Phosphorylation of the NADPH oxidase component p67(PHOX) by ERK2 and P38MAPK: selectivity of phosphorylated sites and existence of an intramolecular regulatory domain in the tetratricopeptide-rich region. Biochemistry. 2003;42(15):4520–6. Epub 2003/04/16. 10.1021/bi0205754 . 12693948

[80] Dang PM, Stensballe A, Boussetta T, Raad H, Dewas C, Kroviarski Y, et al A specific p47phox -serine phosphorylated by convergent MAPKs mediates neutrophil NADPH oxidase priming at inflammatory sites. J Clin Invest. 2006;116(7):2033–43. Epub 2006/06/17. 10.1172/JCI27544 16778989 PMC1479423

[81] Persson C, Carballeira N, Wolf-Watz H, Fallman M. The PTPase YopH inhibits uptake of Yersinia, tyrosine phosphorylation of p130Cas and FAK, and the associated accumulation of these proteins in peripheral focal adhesions. EMBO J. 1997;16(9):2307–18. Epub 1997/05/01. 10.1093/emboj/16.9.2307 9171345 PMC1169832

[82] Fallman M, Andersson K, Hakansson S, Magnusson KE, Stendahl O, Wolf-Watz H. Yersinia pseudotuberculosis inhibits Fc receptor-mediated phagocytosis in J774 cells. Infection and immunity. 1995;63(8):3117–24. 7622239 10.1128/iai.63.8.3117-3124.1995 PMC173425

[83] Black DS, Bliska JB. Identification of p130Cas as a substrate of Yersinia YopH (Yop51), a bacterial protein tyrosine phosphatase that translocates into mammalian cells and targets focal adhesions. EMBO J. 1997;16(10):2730–44. 10.1093/emboj/16.10.2730 9184219 PMC1169883

[84] Black DS, Montagna LG, Zitsmann S, Bliska JB. Identification of an amino-terminal substrate-binding domain in the Yersinia tyrosine phosphatase that is required for efficient recognition of focal adhesion targets. Molecular microbiology. 1998;29(5):1263–74. 10.1046/j.1365-2958.1998.01014.x . 9767593

[85] Alonso A, Bottini N, Bruckner S, Rahmouni S, Williams S, Schoenberger SP, et al Lck dephosphorylation at Tyr-394 and inhibition of T cell antigen receptor signaling by Yersinia phosphatase YopH. J Biol Chem. 2004;279(6):4922–8. 10.1074/jbc.M308978200 . 14623872

[86] Gerke C, Falkow S, Chien YH. The adaptor molecules LAT and SLP-76 are specifically targeted by Yersinia to inhibit T cell activation. The Journal of experimental medicine. 2005;201(3):361–71. 10.1084/jem.20041120 15699071 PMC2213036

[87] Hamid N, Gustavsson A, Andersson K, McGee K, Persson C, Rudd CE, et al YopH dephosphorylates Cas and Fyn-binding protein in macrophages. Microbial pathogenesis. 1999;27(4):231–42. 10.1006/mpat.1999.0301 . 10502464

[88] Gillenius E, Urban CF. The adhesive protein invasin of Yersinia pseudotuberculosis induces neutrophil extracellular traps via beta1 integrins. Microbes Infect. 2015;17(5):327–36. Epub 2015/01/13. 10.1016/j.micinf.2014.12.014 . 25576025

[89] Aepfelbacher M. Modulation of Rho GTPases by type III secretion system translocated effectors of Yersinia. Rev Physiol Biochem Pharmacol. 2004;152:65–77. Epub 2004/09/21. 10.1007/s10254-004-0035-3 . 15378389

[90] Barz C, Abahji TN, Trulzsch K, Heesemann J. The Yersinia Ser/Thr protein kinase YpkA/YopO directly interacts with the small GTPases RhoA and Rac-1. FEBS Lett. 2000;482(1–2):139–43. Epub 2000/10/06. 10.1016/s0014-5793(00)02045-7 . 11018537

[91] Navarro L, Koller A, Nordfelth R, Wolf-Watz H, Taylor S, Dixon JE. Identification of a molecular target for the Yersinia protein kinase A. Mol Cell. 2007;26(4):465–77. Epub 2007/05/29. 10.1016/j.molcel.2007.04.025 . 17531806

[92] Trasak C, Zenner G, Vogel A, Yuksekdag G, Rost R, Haase I, et al Yersinia protein kinase YopO is activated by a novel G-actin binding process. J Biol Chem. 2007;282(4):2268–77. Epub 2006/11/24. 10.1074/jbc.M610071200 . 17121817

[93] Lee WL, Grimes JM, Robinson RC. Yersinia effector YopO uses actin as bait to phosphorylate proteins that regulate actin polymerization. Nat Struct Mol Biol. 2015;22(3):248–55. Epub 2015/02/11. 10.1038/nsmb.2964 25664724 PMC4745138

[94] Groves E, Rittinger K, Amstutz M, Berry S, Holden DW, Cornelis GR, et al Sequestering of Rac by the Yersinia effector YopO blocks Fcgamma receptor-mediated phagocytosis. J Biol Chem. 2010;285(6):4087–98. Epub 2009/11/21. 10.1074/jbc.M109.071035 19926792 PMC2823549

[95] Ke Y, Tan Y, Wei N, Yang F, Yang H, Cao S, et al Yersinia protein kinase A phosphorylates vasodilator-stimulated phosphoprotein to modify the host cytoskeleton. Cell Microbiol. 2015;17(4):473–85. Epub 2014/10/10. 10.1111/cmi.12378 . 25298072

[96] Mohammadi S, Isberg RR. Yersinia pseudotuberculosis virulence determinants invasin, YopE, and YopT modulate RhoG activity and localization. Infection and immunity. 2009;77(11):4771–82. Epub 2009/09/02. 10.1128/IAI.00850-09 19720752 PMC2772528

[97] Kim C, Dinauer MC. Rac2 is an essential regulator of neutrophil nicotinamide adenine dinucleotide phosphate oxidase activation in response to specific signaling pathways. J Immunol. 2001;166(2):1223–32. Epub 2001/01/06. 10.4049/jimmunol.166.2.1223 . 11145705

[98] Kim C, Marchal CC, Penninger J, Dinauer MC. The hemopoietic Rho/Rac guanine nucleotide exchange factor Vav1 regulates N-formyl-methionyl-leucyl-phenylalanine-activated neutrophil functions. J Immunol. 2003;171(8):4425–30. 10.4049/jimmunol.171.8.4425 . 14530369

[99] Yao T, Mecsas J, Healy JI, Falkow S, Chien Y. Suppression of T and B lymphocyte activation by a Yersinia pseudotuberculosis virulence factor, yopH. The Journal of experimental medicine. 1999;190(9):1343–50. Epub 1999/11/02. 10.1084/jem.190.9.1343 10544205 PMC2195683

[100] Condliffe AM, Webb LM, Ferguson GJ, Davidson K, Turner M, Vigorito E, et al RhoG regulates the neutrophil NADPH oxidase. J Immunol. 2006;176(9):5314–20. Epub 2006/04/20. 10.4049/jimmunol.176.9.5314 . 16621998

[101] Amulic B, Cazalet C, Hayes GL, Metzler KD, Zychlinsky A. Neutrophil function: from mechanisms to disease. Annu Rev Immunol. 2012;30:459–89. Epub 2012/01/10. 10.1146/annurev-immunol-020711-074942 . 22224774

[102] Kolaczkowska E, Kubes P. Neutrophil recruitment and function in health and inflammation. Nat Rev Immunol. 2013;13(3):159–75. 10.1038/nri3399 . 23435331

[103] Ley K, Hoffman HM, Kubes P, Cassatella MA, Zychlinsky A, Hedrick CC, et al Neutrophils: New insights and open questions. Sci Immunol. 2018;3(30). Epub 2018/12/12. 10.1126/sciimmunol.aat4579 . 30530726

[104] Mocsai A, Zhang H, Jakus Z, Kitaura J, Kawakami T, Lowell CA. G-protein-coupled receptor signaling in Syk-deficient neutrophils and mast cells. Blood. 2003;101(10):4155–63. Epub 2003/01/18. 10.1182/blood-2002-07-2346 . 12531806

[105] Reinhold A, Reimann S, Reinhold D, Schraven B, Togni M. Expression of SKAP-HOM in DCs is required for an optimal immune response in vivo. J Leukoc Biol. 2009;86(1):61–71. Epub 2009/04/17. 10.1189/jlb.0608344 . 19369640

[106] Eruslanov EB, Singhal S, Albelda SM. Mouse versus Human Neutrophils in Cancer: A Major Knowledge Gap. Trends Cancer. 2017;3(2):149–60. Epub 2017/07/19. 10.1016/j.trecan.2016.12.006 28718445 PMC5518602

[107] Block H, Herter JM, Rossaint J, Stadtmann A, Kliche S, Lowell CA, et al Crucial role of SLP-76 and ADAP for neutrophil recruitment in mouse kidney ischemia-reperfusion injury. The Journal of experimental medicine. 2012;209(2):407–21. Epub 2012/02/01. 10.1084/jem.20111493 22291096 PMC3280874

[108] Green ER, Clark S, Crimmins GT, Mack M, Kumamoto CA, Mecsas J. Fis Is Essential for Yersinia pseudotuberculosis Virulence and Protects against Reactive Oxygen Species Produced by Phagocytic Cells during Infection. PLoS Pathog. 2016;12(9):e1005898 Epub 2016/10/01. 10.1371/journal.ppat.1005898 27689357 PMC5045184

[109] Dahlgren C, Karlsson A, Bylund J. Measurement of respiratory burst products generated by professional phagocytes. Methods in molecular biology. 2007;412:349–63. 10.1007/978-1-59745-467-4_23 . 18453123

[110] Lowell CA, Fumagalli L, Berton G. Deficiency of Src family kinases p59/61hck and p58c-fgr results in defective adhesion-dependent neutrophil functions. J Cell Biol. 1996;133(4):895–910. 10.1083/jcb.133.4.895 8666673 PMC2120842

