# Supplementary figures and images for: Yersinia pseudotuberculosis YopH targets SKAP2-dependent and independent signaling pathways to block neutrophil antimicrobial mechanisms during infection
Source: PLoS Pathog. 2020 May 11;16(5):e1008576. doi: 10.1371/journal.ppat.1008576 (PMC7241846; doi:10.1371/journal.ppat.1008576)

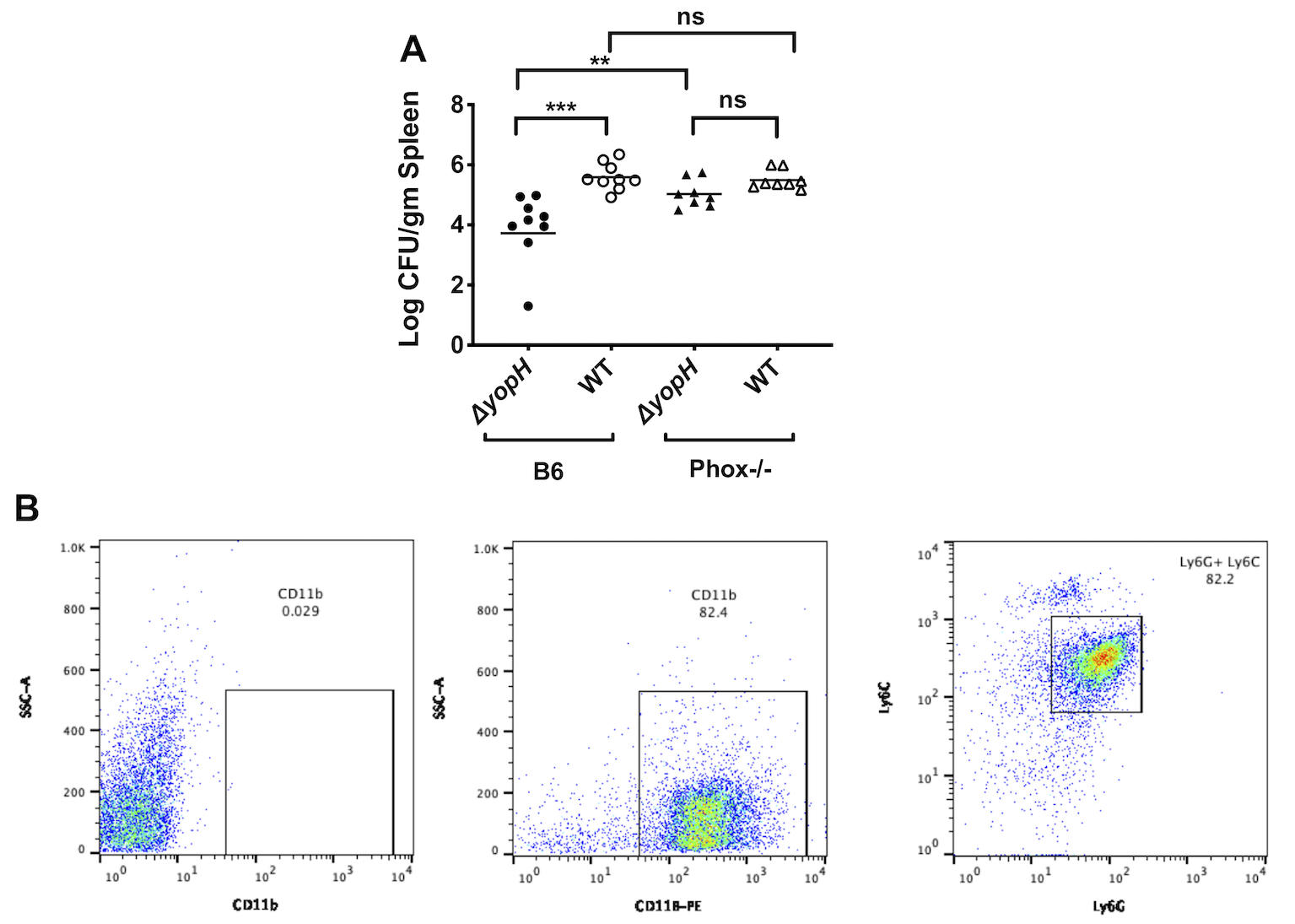

Supplement: S1 Fig — (A) C57BL/6J or C57BL/6J gp91phox-/- mice were I.V. inoculated with a 1:1 mixture of IP2666 WT-Yptb and ΔyopH-KanR. Spleen were collected 2 days post-infection and CFU for WT-Yptb and ΔyopH-KanR was determined by plating on selective and non-selective plates. Statistical significance was calculated using one-way ANOVA with Sidak’s multiple comparison test after log10 transformation of data. (B) Bone marrow was harvested from the tibia and femur bones of mice and neutrophils were isolated using Percoll density gradient method. Cells at the 65–75% interface were collected and stained using with α-CD11b, α-Ly6G and α-Ly6C and analyzed by flow cytometry. Bone marrow isolated neutrophils were more than 80% pure. (TIFF) [file ppat.1008576.s001.tiff]

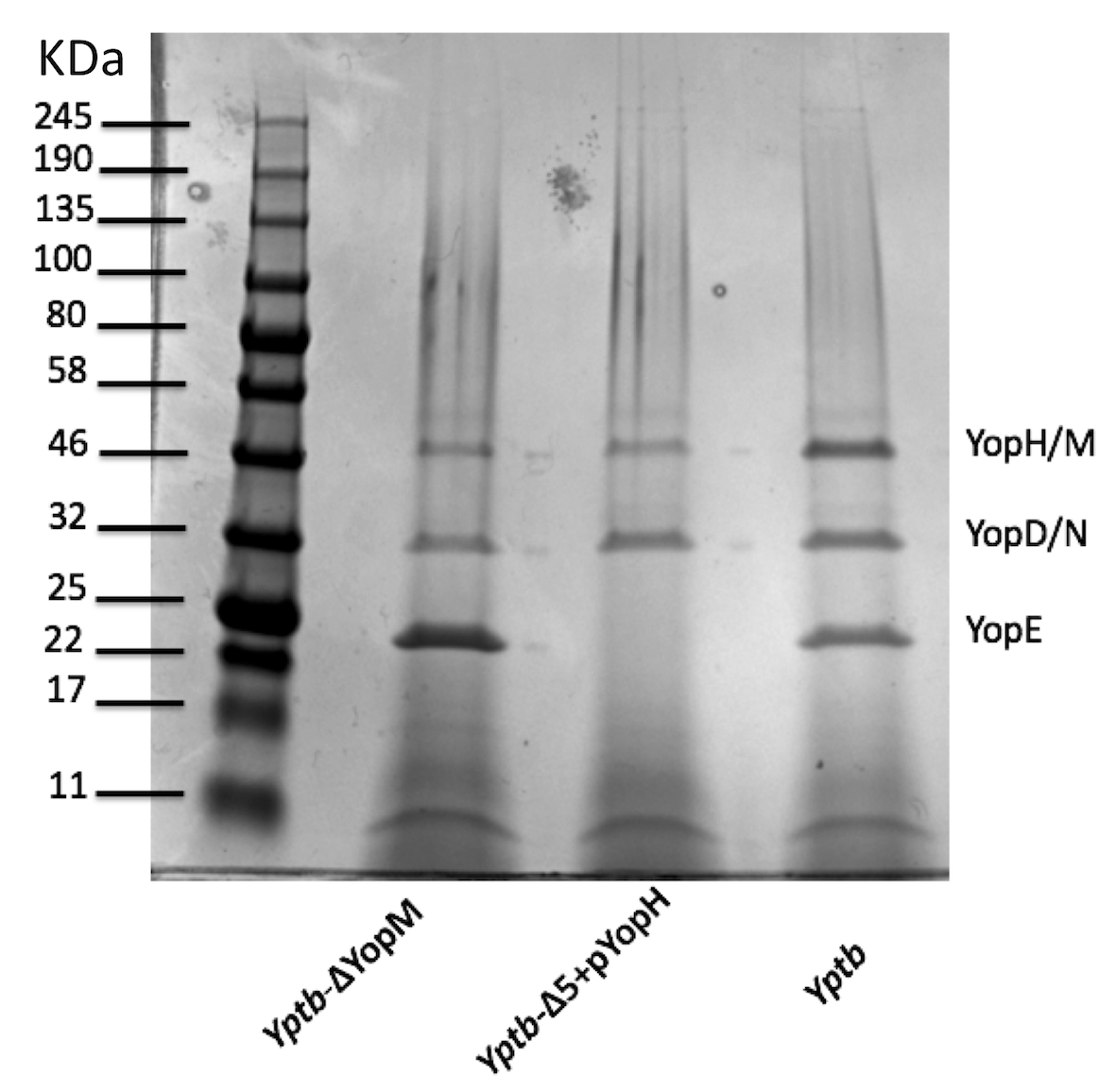

Supplement: S2 Fig — Overnight cultures of IP2666 WT-Yptb, Yptb-Δ5+pYopH and Yptb-ΔyopM were diluted 1:40 in low calcium media and grown for 2 hours at 26°C with aeration followed by addition of 50mM arabinose to induce YopH expression from the pBAD plasmid and shifted to 37°C for 2 hours with aeration. Proteins from culture supernatants were precipitated, resolved on a 4–20% gradient polyacrylamide gel and stained using coomassie blue dye. (TIFF) [file ppat.1008576.s002.tiff]

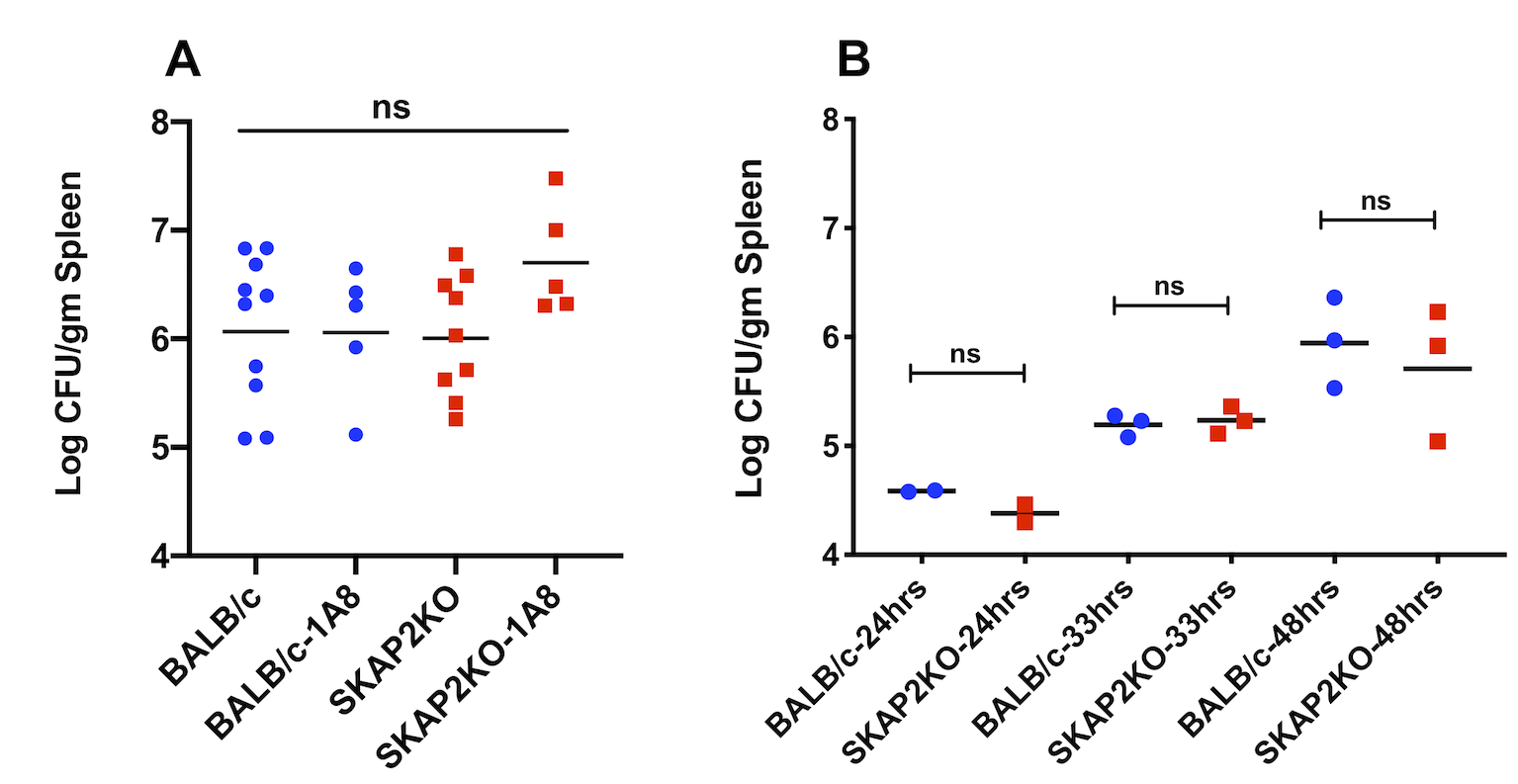

Supplement: S3 Fig — (A) WT-BALB/c and Skap2KO mice were intraperitoneally injected with 1A8 or an isotype control antibody and 16 hrs later inoculated I.V. with an equal mixture of 103 CFU IP2666 WT-Yptb and ΔyopH-KanR. Spleens were collected 3 days post-infection and plated for CFUs. Total CFU are shown. (B)WT-BALB/c and Skap2KO mice were I.V. infected with 1x10^3^ CFU Yptb-GFP, and sacrificed at 24, 33, or 48 hours post-infection. Spleens were collected, weighed, homogenized, and plated for CFU on selective plates. Each dot represents a mouse; horizontal bars represent the geometric mean. Significance was calculated using (A) two-way ANOVA followed by Tukey’s Post-test and (B) Student’s t-test. (TIFF) [file ppat.1008576.s003.tiff]

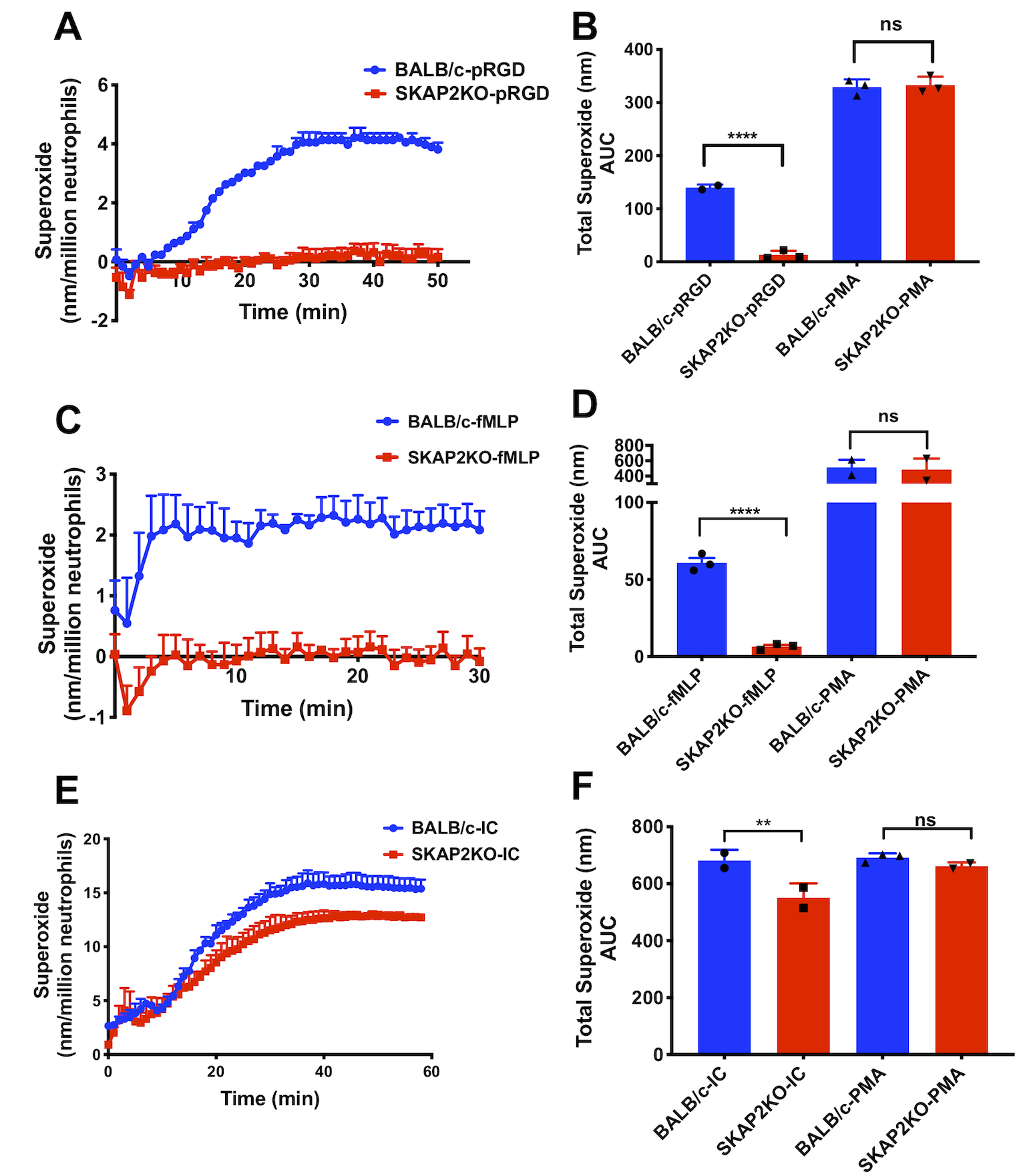

Supplement: S4 Fig — Respiratory burst of WT-BALB/c and Skap2KO BM neutrophils (1x10^5^ cells) was measured using the cytochrome c reduction test by (A-B) plating on a poly-RGD surface, (C-D) priming with 10μg/ml LPS for 20 min followed by stimulation with 1μM fMLP, or (E-F) plating on an IC coated surface. Absorbance at 550 nm and 490 nm was recorded and each condition was corrected by its superoxidase dismutase (SOD) control value. Total amount of ROS was estimated from measuring the AUC for each condition for the duration of the experiment. Data are shown as the means ± SD from triplicate measurements of one experiment, which are representative of at least 4 independent experiments. Statistical significance was calculated using Student t-test. (TIFF) [file ppat.1008576.s004.tiff]

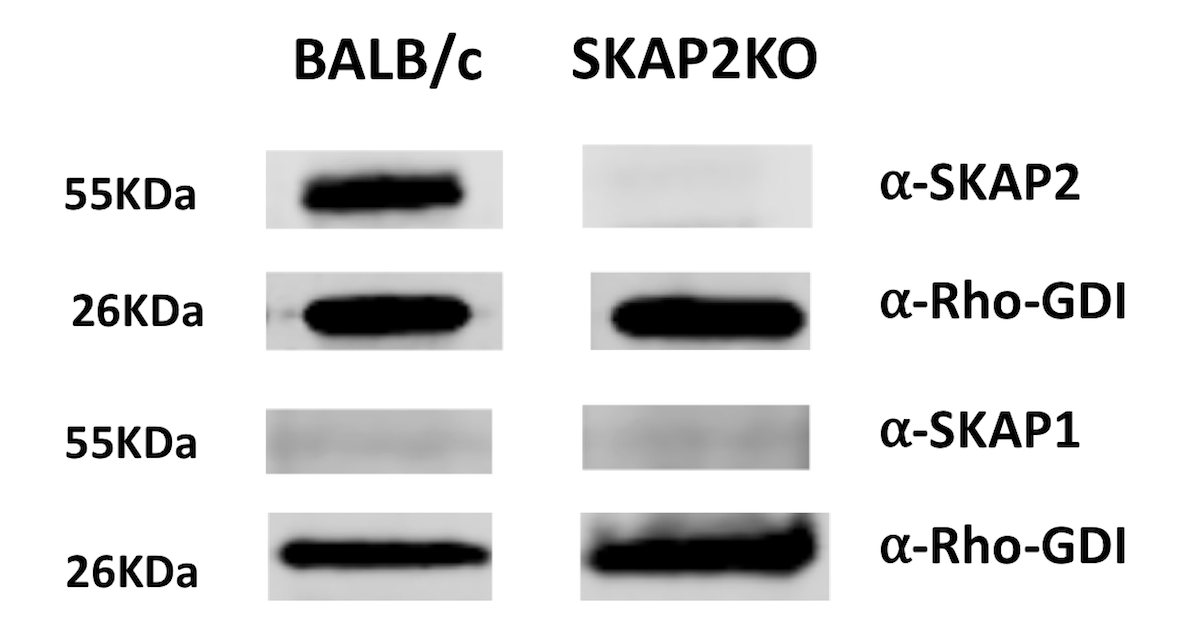

Supplement: S5 Fig — Lysates of BM neutrophils isolated from WT-BALB/c and Skap2KO were immunoblotted for SKAP2 and SKAP1. Anti-Rho-GDI was used as a loading control. No band for SKAP2 was detected in Skap2KO neutrophils confirming the absence of SKAP2. SKAP1 was detected at very low levels in both genotypes. (TIFF) [file ppat.1008576.s005.tiff]

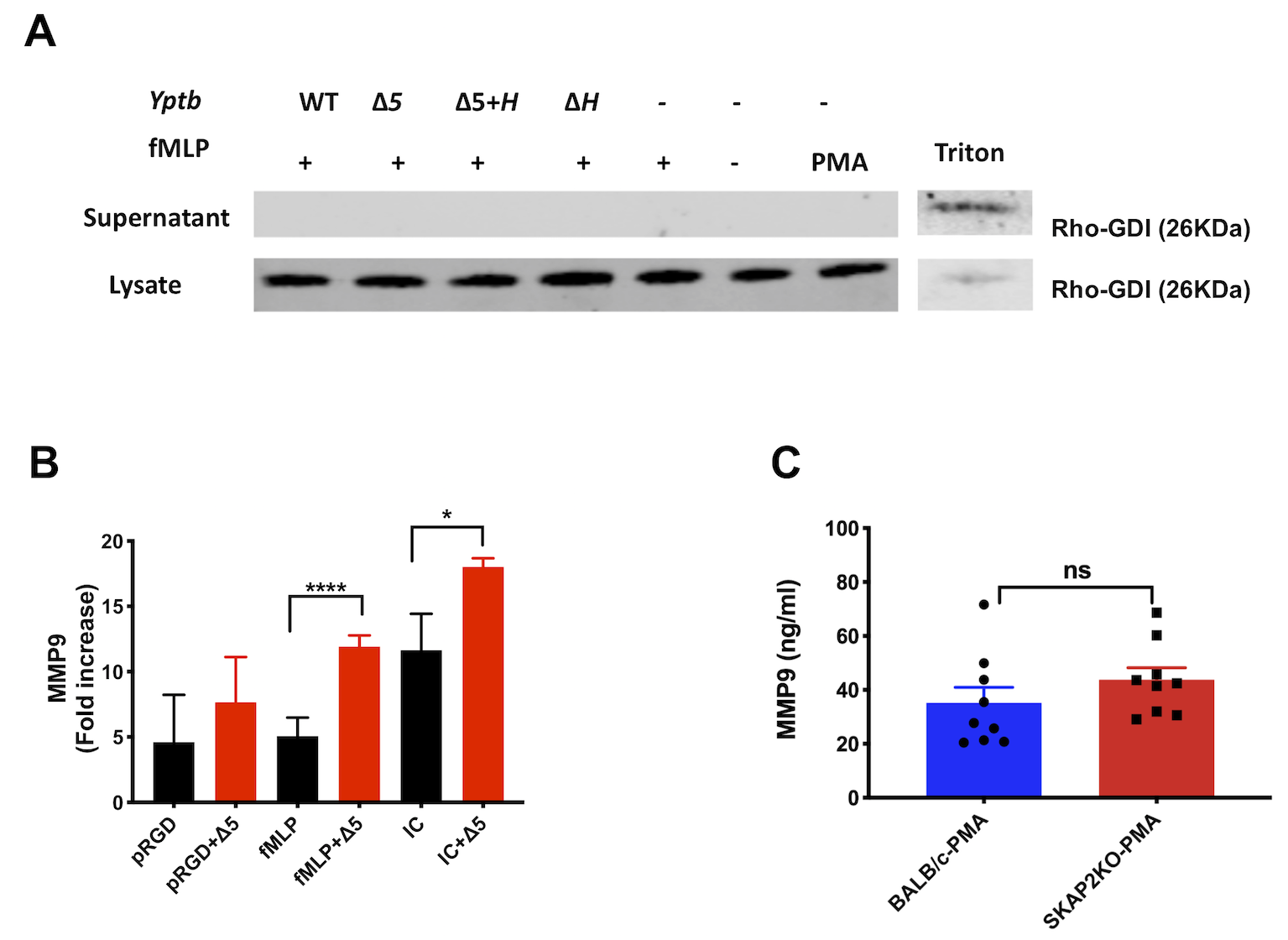

Supplement: S6 Fig — (A) Supernatants and cell lysates (2 x 10^5^ cells) from infected and stimulated samples were immunoblotted with anti-Rho-GDI as an indicator of cell lysis. A positive control for degranulation was treatment with PMA, and the negative control was cells added to FBS-coated wells. Rho-GDI was detected in the supernatants of cells treated with triton X-100. (B) BM neutrophils (2x10^5^) were stimulated with poly-RGD, fMLP or IC to stimulate integrin, GPCR, or FcγR respectively and left uninfected or simultaneously infected with YptbΔ5 at a MOI of 20:1 for 3 hrs. Cell free supernatants from equivalent numbers were analyzed by ELISA for MMP-9 release. Data presented are expressed as fold increase relative to control uninfected and unstimulated samples. The data represent the means ± SEM from 3 independent experiments done in 2–3 replicates. (C) WT-BALB/c and Skap2KO BM neutrophils were stimulated for 3 hrs by 1μM PMA. Cell-free supernatants were analyzed for MMP-9 by ELISA. The data represent the means ± SEM from 3 independent experiments done in triplicate. Statistical significance was calculated using Student t-test. (TIFF) [file ppat.1008576.s006.tiff]
