# Supplementary material for: Yersinia pseudotuberculosis YopH targets SKAP2-dependent and independent signaling pathways to block neutrophil antimicrobial mechanisms during infection
Source: PLoS Pathog. 2020 May 11;16(5):e1008576. doi: 10.1371/journal.ppat.1008576 (PMC7241846; doi:10.1371/journal.ppat.1008576)
Supplement: S1 Table — (DOCX) [file ppat.1008576.s007.docx]

Table S1. Strains used in this study

| Strain  Number | Description | Genotype/ drug resistance | Reference |
| --- | --- | --- | --- |
| LS1  LS2  LS6  LS7  LS27  LS30  LS31  LS32  LS52  LS53  LS54 | *WT-YptbKanR*  *Yptb*Δ*yopH*  *Yptb*Δ*yopHKanR*  *WT-Yptb*  *Yptb+p*GFP  *Yptb+*pBAD  *Yptb*Δ5  *Yptb*Δ5 + pYopH  YPIII*-WT Yptb*  YPIII *Yptb*Δ*yop*H  YPIII *Yptb*Δ*yscF* | IP2666-NdeI-Kan^R^  IP2666 Δ*yopH*-NdeI  IP2666 Δ*yopH*-NdeI-Kan^R^  IP2666-NdeI  IP2666+pACYC184-ptet::gfp  IP2666+ pBAD Cm^R^  IP2666 Δ5+ pBAD Cm^R^  IP2666 Δ5+pBADYopH Cm^R^  WT-YPIIIpIB1  YPIII Δ*yopH*  YPIII Δ*yscF* | (1)  (1)  This work  (2)  (3)  (4)  (4)  (4)  (5)  (5)  (5)  (6) |

1. Fisher ML, Castillo C, Mecsas J. Intranasal inoculation of mice with Yersinia pseudotuberculosis causes a lethal lung infection that is dependent on Yersinia outer proteins and PhoP. Infection and immunity. 2007;75(1):429-42.

2. Ivanov MI, Stuckey JA, Schubert HL, Saper MA, Bliska JB. Two substrate-targeting sites in the Yersinia protein tyrosine phosphatase co-operate to promote bacterial virulence. Molecular microbiology. 2005;55(5):1346-56.

3. Crimmins GT, Mohammadi S, Green ER, Bergman MA, Isberg RR, Mecsas J. Identification of MrtAB, an ABC transporter specifically required for Yersinia pseudotuberculosis to colonize the mesenteric lymph nodes. PLoS Pathog. 2012;8(8):e1002828.

4. Rolan HG, Durand EA, Mecsas J. Identifying Yersinia YopH-targeted signal transduction pathways that impair neutrophil responses during in vivo murine infection. Cell Host Microbe. 2013;14(3):306-17.

5. Logsdon LK, Mecsas J. Requirement of the Yersinia pseudotuberculosis effectors YopH and YopE in colonization and persistence in intestinal and lymph tissues. Infection and immunity. 2003;71(8):4595-607.

6. Davis AJ, Mecsas J. Mutations in the Yersinia pseudotuberculosis type III secretion system needle protein, YscF, that specifically abrogate effector translocation into host cells. J Bacteriol. 2007;189(1):83-97.
